# Transcriptomic profiling of human brown and white adipose tissue depots

**DOI:** 10.1038/s41467-026-76065-7

**Published:** 2026-07-29

**Authors:** Nhuquynh Nguyen, Cheryl Cero, Adrienne R. Guarnieri, Ju Hee Kim, Kelly T. Long, Jurgen Heymann, Sahara L. Ali, Emily V. Leary, Aaron M. Cypess

**Affiliations:** 1 Diabetes, Endocrinology, and Obesity Branch, National Institute of Diabetes and Digestive and Kidney Diseases (NIDDK), NIH, Bethesda, Maryland USA; 2 Biostatistics Program, National Institute of Diabetes and Digestive and Kidney Diseases (NIDDK), NIH, Bethesda, Maryland USA

**Keywords:** Fat metabolism, Obesity

## Abstract

Human adipose tissues are solid organs distributed throughout the body in specific locations and are thought to have dichotomous functions: white adipose tissue stores triglycerides as an energy reservoir, and brown adipose tissue uses metabolic fuels to generate heat. To define the transcriptional profile of six principal adipose depots, we analyze tissue from 11 autopsies (3 F/8 M, 16-71 y) of patients who had different underlying diseases. Despite global similarities in terms of cell type proportions, each depot has a distinct transcriptomic signature and functional profile. Each human’s adipose tissues are more similar to each other than to the anatomically and functionally matching depots in other patients. The transcriptomic pattern of subcutaneous white adipose tissue can even be used to predict the human donor. This pattern suggests that individualized factors such as genetics, age, environment, diet, and disease play impactful roles in coordinating the behavior of the whole-body adipose tissue depot.

## Abstract (web summary)

White and brown adipose tissues play opposing roles in energy storage and expenditure. Here, the authors show that their transcriptional profiles are more similar within an individual than between anatomically and functionally equivalent depots across different individuals.

## Introduction

The body’s adipose tissue (AT) depots play central roles in metabolism and reproduction. White adipose tissue (WAT) depots store triglycerides, and brown adipose tissue (BAT) depots, more localized and much smaller in mass, specialize in energy expenditure. With the recognition that WAT and BAT are physiologically active organs in humans of all ages, capable of regulating processes as wide-ranging as energy balance and inflammation^1^, new questions about adipose tissue structure and function have taken center stage: do the different depots have distinct functions, and how much functional intradepot coordination occurs? Prior studies of human AT have typically used surgical specimens that, by necessity, were restricted only to two to three different sites at most, and some depots have never been analyzed due to their inaccessibility. In this work, we sought to gain as comprehensive a dataset as possible to address these questions.

## Results

### Bulk RNA-seq transcriptomics of human adipose tissue depots

**Fig. 1:**
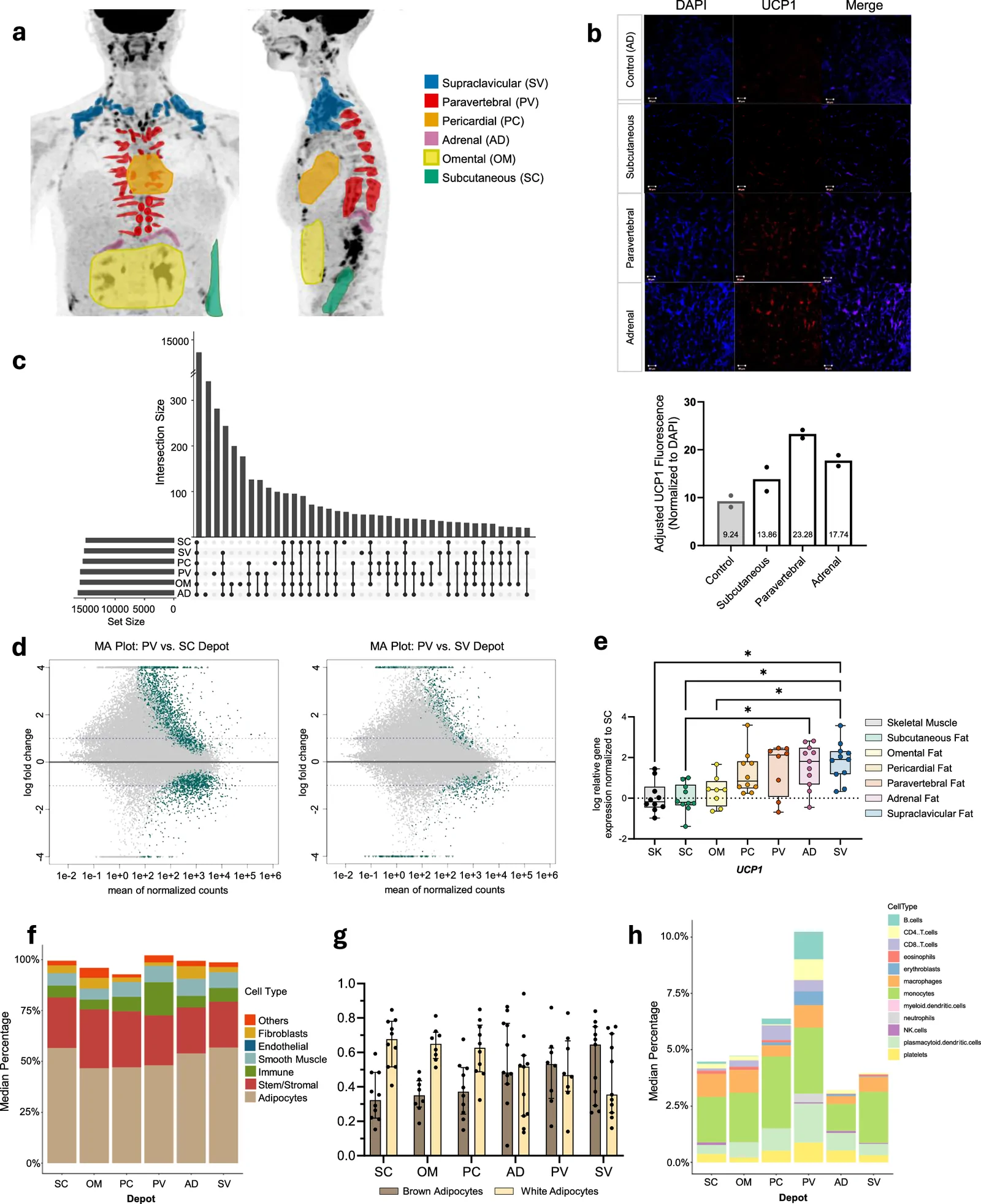
Multimodal characterization of human adipose depots: imaging, gene expression, and cellular composition. **a** ^18^FDG-PET images, frontal (left) and sagittal (right), of a healthy female subject (23 y), with each depot labeled: subcutaneous (SC), omental (OM), pericardial (PC), paravertebral (PV), adrenal (AD), and supraclavicular (SV). **b** UCP1 immunofluorescence in SC, PV, and AD depots. UCP1 immunofluorescence in SC, PV, and AD depots. UCP1 fluorescence intensity was normalized to DAPI for each sample. Data represent the average normalized UCP1 fluorescence from two experimental sessions (*n* = 2) for one subject, with the AD depot used as the no-primary-antibody control. Scale bar depicts 50 µm. **c** UpSet plot showing the number of genes expressed in each depot (PC, AD, OM, PV, SC, and SV) and their overlaps. Using log-transformed, normalized counts per million (CPM) across all depots, a gene was considered expressed in a specific depot if its trimmed mean of M values (TMM) normalized, log-transformed CPM exceeded the threshold of 0. **d** MA plots visualizing the differential expression: between PV vs. SC (left) and PV vs. SV (right). Significance was calculated using a two-sided Wald test via DESeq2, with multiple testing adjustments applied using the Benjamini-Hochberg method (FDR < 0.05). Genes with a statistically significant adjusted *P*-value (< 0.05) are highlighted in teal. **e** qPCR analysis of *UCP1* (a thermogenic marker) across all fat depots and skeletal muscle, SK, (as a negative control). Gene expression data were normalized to β-actin and the SC depot, then log-transformed and analyzed using a mixed-effects analysis. Each point is an independent biological replicate. The center line represents the median, the box bounds represent the 25th and 75th percentiles, and the whiskers indicate the minimum and maximum values. * = *p* < 0.05, Tukey’s multiple comparison test, two-sided. Individual *p-*values are SK vs SV, *p* = 0.038; SC vs SV, *p* = 0.016; SC vs AD, *p* = 0.026; OM vs SV, *p* = 0.043. *n* = 10 (SK), 10 (SC), 8 (OM), 10 (PC), 8 (PV), 11 (AD), 11 (SV). **f** Cellular deconvolution of bulk transcriptomic data showing the median percentages of adipocytes, stem/stromal cells, immune cells, smooth muscle cells, endothelial cells, and other cell types across depots. Percentages lower than 5% are not labeled. **g** Cellular deconvolution of bulk transcriptomic data showing the median proportions of brown versus white adipocytes. The height represents the median, and the whiskers indicate the 25th and 75th percentiles. Each point is an independent biological replicate. *N* = 10 (SC), 8 (OM), 10 (PC), 11 (AD), 8 (PV), 11 (SV). **h** Cellular deconvolution of immune cells, including B cells, CD4 + T cells, CD8 + T cells, eosinophils, erythroblasts, macrophages, monocytes, neutrophils, NK cells, plasmacytoid dendritic cells, and platelets. Values are median and IQR percentages. Percentages lower than 0.5% are not labeled. Source data are provided as a Source Data file.

We analyzed AT from 11 autopsy cases (3 F/8 M, mean age: 48.6 ± 6.1 years (SD)). The majority (*n* = 10) of patients’ deaths were from malignancies; and one was from an immune-related disorder (Supplementary Table 1 and Supplementary Data 1). Adipose tissues came from six defined anatomical locations (Fig. 1a): abdominal subcutaneous (SC), omental (OM), pericardial (PC), periadrenal (AD), paravertebral (PV), and supraclavicular (SV). In addition, we procured urinary bladder (UB) and skeletal muscle (SK) to serve as non-AT controls. Tissue was fixed and analyzed via immunofluorescence for uncoupling protein 1 (UCP1), the defining protein of thermogenic adipocytes. As expected, the SC tissue showed minimal levels of UCP1 and also of tyrosine hydroxylase (TH), which reflects sympathetic innervation. In contrast, both AD and PV regions displayed similarly higher expression of both genes (Fig. 1b). We also examined the expression of other thermogenic genes across the depots and found that each demonstrated a gradient that varied in different degrees from what we observed with *UCP1* (Supplementary Fig. 1b–d). This pattern is consistent with previous reports of brown adipocyte areas within AD adipose tissue^2^ and indicates that the PV depot, which has not yet been described due to its surgical inaccessibility, can be categorized as BAT.

To establish a transcriptomic signature of each of the six human AT depots, we performed bulk RNA-seq analysis and generated an UpSet plot (Fig. 1c and Supplementary Data 2). A prior analysis using microarrays of gene expression differences between 15 different human adipose tissue depots found that even after distinguishing subcutaneous and visceral depots, each specific site had a distinct expression pattern that reflected differences in metabolism, immunity, and physical function^3^. Analogously, we found that 14,202 genes were commonly expressed across all depots. Each adipose depot displayed its own distinct transcriptomic signature: SC expressed 55 unique genes; OM 177; PC 99; PV 282; AD 342; and SV 49 (Supplementary Data 3). To quantify the magnitude of these differences, we visualized each comparison with MA plots (Fig. 1d and Supplementary Fig. 2), which revealed distinct modes of transcriptional regulation between different depots. The comparison between PV and SC was defined by a larger number of genes with high-magnitude fold-changes, many of which were asymmetrically upregulated in PV tissue. In contrast, the differences between PV and SV were characterized by less high-magnitude fold-changes.

Consistent with previous work^4^, qPCR analysis revealed a gradient of increasing UCP1 expression: SK = SC < OM < PC < PV < AD < SV (Fig. 1e), where SK served as the negative control. Preliminary exploratory deconvolution analysis (Supplementary Data 4) showed that adipocytes were the most abundant single cell type across all AT depots (Fig. 1f)^5^. Additional layers of deconvolution showed that the proportion of brown versus white adipocytes follows the pattern observed from the qPCR (Fig. 1g and Supplementary Data 5)^6^. The percentage of adipocytes across depots ranged from 40–60%, which was in between those previously reported^5,7^. The proportions and compositions of immune cells^5^ (Fig. 1h) was similar to a recent study of cells derived from human neck AT^8^. We then applied one further layer of cellular deconvolution to the dataset to determine whether any of these subtypes are enriched in specific adipose depots and whether their distribution correlates with covariates age, sex, and cause of death. We used a gene signature set that included structural Wnt-regulated adipose tissue-resident (SWAT), adipogenic, and progenitor cell types (Supplementary Fig. 3)^9^. All the adipose depots had similar proportions of SWAT (*p* = 0.942) and adipogenic (*p* = 0.099) cells as assessed by single-factor ANOVA. Progenitor cells were the only ones that significantly differed, with a single-factor ANOVA (*p* = 0.029).

### Gene ontology analysis of AT depots

**Fig. 2:**
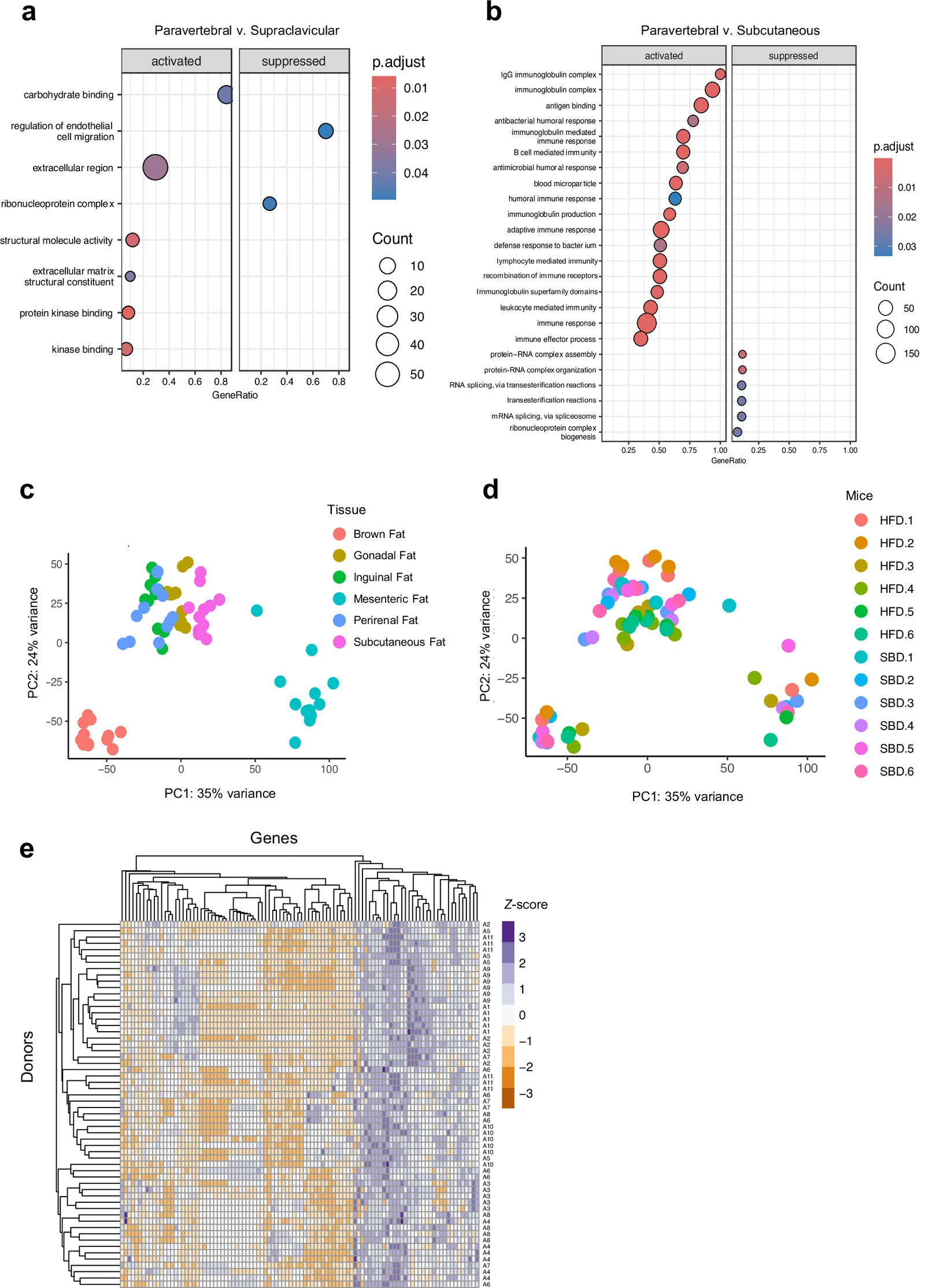
Gene ontology of human white and brown adipose tissues and mouse adipose tissue transcriptomics. **a**, **b** Dot plots showing representative pathways from gene ontology (GO) analysis of enriched genes in the (**a**) PV depot compared to the SV depot and the (**b**) PV depot compared to the SC depot. Significance was calculated via Gene Set Enrichment Analysis (GSEA) using 10,000 permutations to assess both positive and negative enrichment. Multiple testing adjustments were applied using the False Discovery Rate (FDR) method (FDR < 0.05). Two-sided comparisons were used. **c**, **d** Principal component analysis (PCA) of mouse microarray data based on differential gene expression (DGE) analysis shows clustering by (**c**) six adipose tissue depots—interscapular BAT, perigonadal WAT, inguinal WAT, mesenteric WAT, perirenal WAT, and subcutaneous WAT—compared to (d) clustering by individual mice under different dietary conditions. **e** Hierarchically clustered heatmap of (**top**) human genes and (**left side**) donors using average linkage clustering generated from the top 100 most variable genes across all depots after variance stabilizing transformation. The color scale represents *Z*-scores. Source data are provided as a Source Data file.

To further understand the physiological implications of these findings, we performed a gene ontology (GO) analysis^10^ to determine how the AT depots compared to each other (Fig. 2a and Supplementary Fig. 4). SV is widely recognized as the predominant human BAT depot^11^ and is associated with enhanced cardiometabolic health^12^. PV exhibited only eight significantly enriched pathways compared to SV, indicating that PV is transcriptomically and likely functionally similar to SV. These data suggest that the PV depot, which can vary from the smallest to the largest BAT depot in humans^13^, may also be important for cardiometabolic health. Compared to the SC depot (Fig. 2b), PV included activated pathways related to B cell immunity^14^ and signaling, which aligns with our immune cell deconvolution analysis.

### Clustering of mouse and human tissue transcriptomics

Given the functional similarities between human and mouse adipose tissues^15,16^, we expected to observe similar transcriptomic patterns across depots in both species. Differential gene expression (DGE) analysis of published mouse microarray data^17^ revealed a distinct clustering pattern via principal component analysis (PCA): the clusters were much closer together when grouped by anatomical depot compared to grouping by individual mouse (Fig. 2c, d and Supplementary Data 6). We quantified the proximity of PCA cluster points using a modified Calinski-Harabasz Index (CH-I). A higher CH-I indicates a compact cluster that is also spatially distant from other clusters, while a CH-I closer to 0 indicates a dispersed spatial patterning^18^. The median CH-I among points grouped by mice AT depot was 3.999 (IQR 35.346) while the median CH-I when grouped by individual mice was 0.283 (IQR 0.391) (*p* = 0.0001) (Supplementary Table 2). This difference indicates that a specific depot had a gene expression signature that was more similar to the same depot in other mice than it had with other depots within the same mouse. This finding was further supported by variance partitioning analysis (Supplementary Fig. 5a and Supplementary Data 7), which showed that mouse identity only accounted for an average of 18.6% of the gene variability while tissue identity accounted for 56.8% (*p* < 2.2e-16).

In contrast, when applying the same DGE analysis and PCA to human autopsy sample data, the opposite clustering pattern emerged. A hierarchically clustered heatmap based on gene expression levels in each donor and depot using average linkage^19^ grouped tissues together predominantly by patient rather than by depot, with particularly close clustering seen for patients A1, A3, A4, A9, and A10 (Fig. 2e). To corroborate this observation, we plotted the DGE results via PCA, analogous to what was done with the mouse tissue in Fig. 2b, and again found the opposite of what was seen from the mice—points grouped by donor clustered much more closely than those grouped by depots, both visually and quantitatively (Fig. 2d, e and Supplementary Table 3). The median CH-I by depots was 0.605 (IQR 0.759), whereas for the donors, it was 3.329 (IQR 4.568) (*p* = 0.007). This was further supported by variance partitioning analysis, which showed that donor identity accounted for a substantially greater proportion of gene expression variance (mean = 37.6%) compared to depot (mean = 5.4%, *p* < 2.2e-16), reinforcing the observation that gene expression clusters more strongly by individual than by anatomical location in human adipose tissue (Supplementary Fig. 5b and Supplementary Data 8). We performed a variance-partitioning analysis on data from the top 5000 differentially expressed genes (as determined by PCA) with only donor or depot identity as the random effects to first quantify the variability without the following potential covariates: age, sex, postmortem interval (PMI), BMI, and RIN. We then added the covariates and found that donors explained on average 37.5% of the variance across all 5000 genes, while depots only explained 5.4% (*p* < 2.2e-16 under Mann Whitney-U) in the model with only depots and donors as random effects. When the other covariates were added, we found that while BMI contributed 15.1% of the gene variance, donors still accounted for slightly more variance at 15.6% (*p* = 0.155). No other covariate came close to explaining as much gene variance (Supplementary Fig. 5c and Supplementary Data 9). In summary, for mouse adipose tissue, intradepot variability was lower than intrasubject variability, but in humans, it was the opposite: intrasubject variability was lower than intradepot variability.

**Fig. 3:**
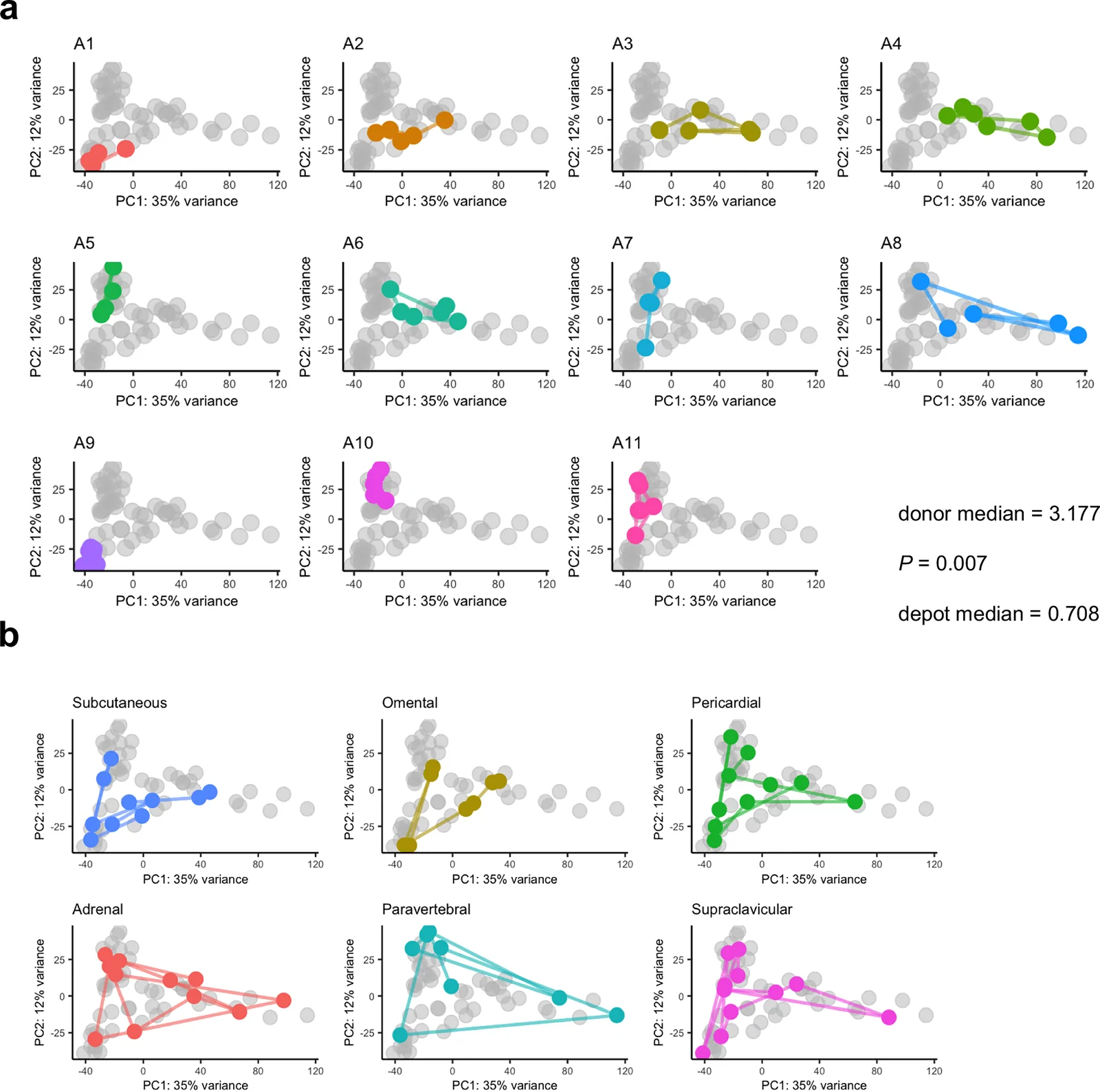
Comparison of intrasubject and intradepot transcriptomic variability in humans. **a** PCA was performed on variance-stabilized counts to explore sample variance across depots for each donor. Lines connecting the points represent Euclidean distances. Euclidean distances between points within the two-dimensional PCA space (PC1 and PC2) were calculated geometrically to quantify sample similarity; no statistical hypothesis testing was applied to these distances. Two-sided comparisons were made. **b** PCA of DGE results across donors for each depot. Lines connecting the points represent Euclidean distances. Source data are provided as a Source Data file.

To further explore the robustness of our initial findings, we re-examined the variability using multiple different gene subsets. Our initial analysis included the top 5000 genes (Fig. 3a, b). We then included only adipocyte-related genes^20,21^, and the pattern persisted, with depots showing a median CH-I of 0.708 (IQR 0.868) and donors 3.177 (IQR 5.402) (*P* = 0.012) (Supplementary Fig. 6a). Given that immune-related genes can be differentially expressed in several tissues post-mortem, we excluded immune genes^22^ (Supplementary Fig. 6b), yet the intradepot variability remained higher, with a median depot CH-I of 0.469 (IQR 0.482) and a median donor CH-I of 3.505 (IQR 2.726) (*p* = 6.46E-04). We determine the influence of PMI on gene expression by using a published dataset describing the gene expression patterns associated with PMI in various human tissues^23^ (Supplementary Datasheet 9). The PMI-associated genes identified in adipose tissue did not correlate significantly with the PMI in adipose depots in our cohort. These results suggest that immune genes do not significantly correlate with PMI, and we would therefore not expect them to confound the depot study results. Rather, in a total of 11 different gene subsets, the pattern of lower intrasubject variability compared to intradepot variability was consistently significant (Supplementary Table 4). We also found that donors within 0.5 standard deviations from the mean age range exhibited the least variability, clustering most tightly (Supplementary Fig. 6c). Finally, we more closely matched the mouse and human cohorts by removing the female human donors (Donors A3, A7, and A8) and any human donors whose cause of death was not a malignancy (Donors A3 and A7). The clustering analysis followed the same pattern (Supplementary Fig. 7). Studies have shown that long noncoding RNAs (lncRNAs) define tissue types better that the coding genes^24^. In addition, lncRNAs have been described as regulators of metabolic processes in adipose tissue^25^. We performed a separate analysis of noncoding RNAs to include or exclude all lncRNA genes (Supplementary Fig. 8a–d). Either way, donors clustered more closely than depots. To investigate whether depot-specific clustering could be resolved by accounting for inter-individual variability, we implemented a batch correction strategy. Since covariates such as age and sex are inherent, fixed properties of each donor, their effects are nested within the overall donor effect and are accounted for in this correction. The resulting PCA plot shows that even after accounting for donor-specific signatures, clear depot-specific clusters do not emerge (Supplementary Fig. 8e). This result strengthens our primary conclusion that profound inter-individual differences are the largest driver of transcriptomic variation in this dataset, outweighing the variation observed between different adipose depots. The combination of multiple different analyses confirms that the general pattern holds across a variety of gene sets: intradepot variability was greater than intrasubject variability.

The relationship between intrasubject and intradepot variability was reversed under specific conditions. For instance, when we partitioned the dataset for genes which demonstrate the most variability across depots, we observed the exception: the intradepot variability was lower than intrasubject variability. Using this set of 47 genes (Supplementary Data 10), donors had a median CH-I of 0.377 (IQR 1.078) while depots had a median CH-I of 3.496 (IQR 5.850) (*p* = 0.005) (Supplementary Fig. 9a). The genes in this set could serve as a refined set of molecular signatures to define distinct adipose depots. Among these was *TBX15*, which has already been shown to distinguish human AT depots both structurally and functionally^26,27^.

### Transcriptomic comparison of adipose tissues to bladder

The distinct clustering patterns observed in adipose depots led us to investigate whether gene expression was globally coordinated across other, non-adipose tissues in the autopsy subjects. To explore this, we used the urinary bladder (UB) as a non-adipose control and performed a centroid analysis. UB samples were significantly farther from the fat depot centroid (average distance: 58.75 ± 27.94) compared to adipose tissue samples (36.85 ± 25.47; *P* = 0.003). Similarly, when comparing distances to the UB centroid, adipose tissue samples were farther away (66.45 ± 23.80) relative to UB samples (31.18 ± 15.11; *P* = 7e-06) (Supplementary Fig. 9b). We further investigated the natural grouping of the UB by performing unsupervised k-clustering with three specified clusters (Supplementary Fig. 9c). Half of the UB samples formed their own distinct cluster, while the remaining UB samples were positioned at the periphery of clusters dominated by adipose tissues. These findings indicate that adipose depots cluster more closely with each other than with UB, even within the same individual. This suggests that the AT clustering patterns were driven by their shared transcriptomic characteristics rather than being an artifact of the PCA.

### Donor prediction based on subcutaneous WAT transcriptomics

**Fig. 4:**
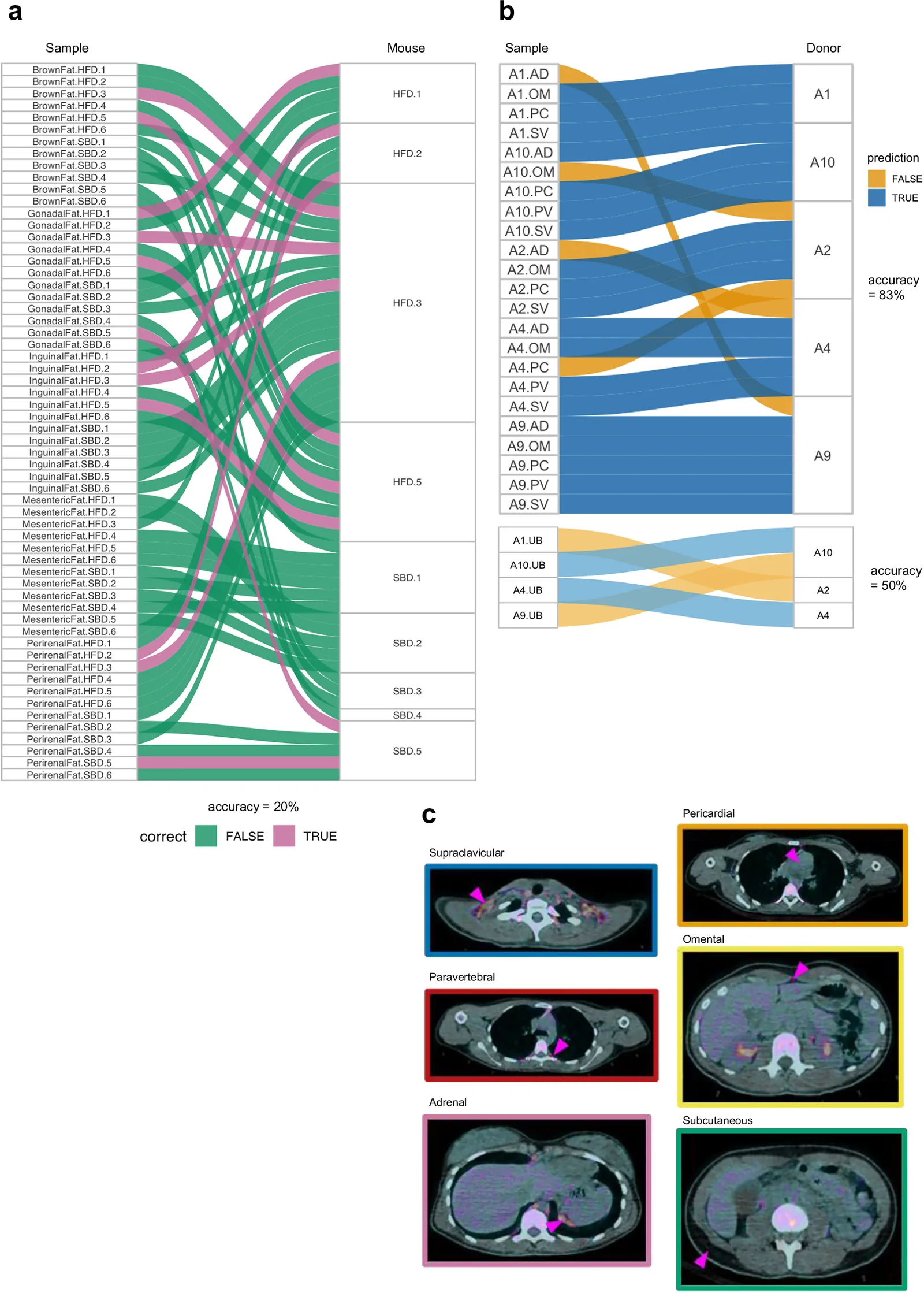
Predictive modeling of mouse and human donors and functional corroboration of human adipose tissue function. **a** Sankey diagram of a Random Forest analysis of microarray data using as the training set the inguinal WAT depot from 12 wk-old DBA/2 J mice fed 6 wk with either standard breeding diet (SBD) or high-fat diet (HFD). The model achieved 20% accuracy (pink = true; green = false) in predicting whether the sample originated from the same mouse. **b** Sankey diagram of a Random Forest analysis using (**top**) the human subcutaneous WAT depots as the training dataset and considering adipose samples only. The model achieved 83% accuracy in predicting the donor identity for autopsy tissues from donors 1, 4, 9, and 10. When the urinary bladder (**bottom**) was included in the model, the accuracy was 50%. **c** Axial images of six human adipose tissue depots in a 23 y woman after pharmacological stimulation of WAT and BAT metabolic activity as measured by ^18^F-FDG PET/CT. Source data are provided as a Source Data file.

This intradepot similarity has clinical implications, as the principal challenge to studying the cellular and molecular characteristics of most human AT depots is that they are poorly accessible except via invasive surgery or autopsy. To determine if we could predict any particular AT depot’s original donor, we used the generally accessible SC depot as the training dataset in a Random Forest model. For the mouse, prediction of the original animal was only 20% accurate (Fig. 4a) but was 75% accurate when predicting the tissue origin (Supplementary Fig. 10a). In contrast, the human pattern was the inverse: there was 83% accuracy in predicting the donor (Fig. 4b), but only 20% when attempting to identify the original depot (Supplementary Fig. 10b). As expected, the donor’s UB was distinct from the AT: one could predict the transcriptional profile of other UB’s but not the original patient. To confirm that the predictive signal is biologically meaningful, we trained a model on SC samples and tested it on withheld visceral (omental) samples. The model achieved 80% accuracy. In contrast, a control model trained on randomized donor labels achieved an accuracy of 20%, confirming the model is learning a true, donor-specific biological signal (Supplementary Fig. 11).

The comparatively lower transcriptomic variability in the mouse dataset compared to the humans studied here could arise from several drivers leading to essential differences in the datasets. The mice were genetically identical; had the same environments in terms of housing and temperature; consumed one of two defined diets; lived the same number of weeks when the interventions began; and remained otherwise healthy. These features combined will minimize inter-individual variability and have been shown to affect mouse transcriptomics^28^. Not one of these factors is applicable to the subjects studied here or humans in general, who are genetically, clinically, and environmentally heterogeneous in both known and unknown ways. Nevertheless, a value of this comparison is that inbred mice are often used to understand the behavior of analogous human tissues, so the similarities and differences identified here will be supportive in refining models of adipose tissue physiology. It will be informative to see how variations in these factors in outbred or wild mice affect their adipose tissue transcriptomics when compared to both inbred mice and to humans.

### Corroboration of transcriptomics using functional imaging

We investigated if the transcriptomic profiling in the 11 donor patients was corroborated functionally in a cohort of living, healthy human subjects whose adipose tissue metabolic activity was measured in real-time using ^18^F-FDG PET/CT^29^. SUVmax reflects the highest level of glucose uptake in a depot^11^. Among 14 healthy women, we observed two distinct categories of human adipose tissues in response to adrenergic stimulation: the SV, PV, and AD depots had high glucose uptake, with a mean SUVmax of 25.56 ± 20.71. In contrast, OM, PC, and SC depots all had lower uptake, with a mean SUVmax of 0.70 ± 0.31 (*p* = 1.41E-09) (Fig. 4c). Thils pattern supports classifying SV, PV, and AD as thermogenic tissues, consistent with their higher *UCP1* levels in the autopsy-based dataset (Fig. 1b). In contrast, OM, PC, and SC had far lower glucose uptake, further affirming their classification as WAT. This corroboration of gene expression and tissue glucose uptake suggests that other functional features identified by the transcriptomic profiling of autopsy tissue might be seen in the general population of healthy adults.

## Discussion

This study is limited by several factors. The heterogeneity of the human cohort may have led to an identification of correlations that were specific to the distinct group, while at the same time, missed physiological patterns that would have been found within a larger cohort. The use of autopsy tissue may have brought variations in post-mortem interval and differential degradation of RNA, which could have affected the transcriptomic profiles. Therefore, the small sample size and specific population reduce the generalizability of the findings. On the analytical side, several exploratory tools were used, such as cellular deconvolution, the modified CH-Index, and Random Forest classification. These carry the risk of bias and limited generalizability. For example, cell deconvolution of bulk RNA-seq data can be impacted by the accuracy and generality of cell signature assignments offered by the applied reference data. As this field rapidly evolves and single-cell human tissue atlases continue to grow, we anticipate that comprehensive adipose tissue references and improved algorithms for bulk RNA-seq deconvolution will become available. Larger and functional studies are needed to directly test the physiological relevance of the transcriptomic profiling reported here.

In conclusion, we identified distinct characteristics of the transcriptomic profiles human patient AT that have potentially broad physiological ramifications. We show that each of the six principal human adipose depots is a distinct organ with a unique transcriptomic signature reflecting overlapping yet discrete functions. Nevertheless, the BAT and WAT depots in any single human are more like each other at the level of gene expression than the anatomically and functionally matching depots in a different person. This pattern suggests that individualized factors such as genetics, age, environment, diet, and disease play impactful roles in coordinating the behavior of the whole-body adipose tissue depot. Mechanistically, the dynamic influences could include newly discovered adipokines^30^ that orchestrate this coordinated activity. Given the crosstalk seen among AT and other mesoderm-derived tissue^31^, it is intriguing to speculate if organs analogous to adipose tissue in terms of anatomical distribution and distinct function, such as skeletal muscle and bone, also share this personalized transcriptomic coordination. Ultimately, it remains to be determined whether these patterns will be seen in healthy humans and how they evolve in the setting of pathological metabolic and other disease states.

## Methods

### Research involving human volunteers and human tissue

The research presented here complies with all relevant ethical regulations. This study used two independent human datasets, described in detail below. Imaging data were obtained from an ongoing clinical trial (NCT 03049462) that consists of three cohorts that are independent. Cohort 1 (used here) comprises 14 healthy women, and its primary endpoint is published in ref. ^29^. The clinical trial data presented here were collected at baseline prior to any intervention. The second dataset consists of transcriptomic data that were obtained from tissue collected at autopsy at the NIH Clinical Center.

### Study approval

The clinical trial that studied 14 healthy women who underwent ^18^F-FDG PET/CT imaging was registered with Clinicaltrials.gov (NCT03049462) and has the FDA Investigational New Drug registration number 116246. Sex was considered in the study design and was determined based on self-reporting. This study was approved by the Human Studies Institutional Review Board of the NIDDK and the NIH Radiation Safety Committee. Healthy volunteers were recruited by word of mouth or through the Patient Recruitment and Public Liaison Office of the Clinical Center. The volunteers provided written informed consent according to the Declaration of Helsinki principles and were compensated.

### Collection of human adipose tissue samples

The adipose and bladder tissue samples used for the transcriptomic studies were collected from autopsies. This procurement was not part of a clinical trial and was done with the support of the Pathology Core at the National Cancer Institute at the NIH. As confirmed by the NIH Clinical Center and IRB guidelines, per the NIH policy, because all of the samples were acquired from deceased individuals, the study is not considered human subjects research. Therefore, consent was waived by the IRB. This policy is based on United States Department of Health and Human Services human subject regulations under 45 CFR 46, wherein a human subject is defined as “a living individual about whom an investigator (whether professional or student) conducting research obtains (1) data through intervention or interaction with the individual or (2) identifiable private information. Sex was not considered in the design. Sample size was not considered in the design. Rather, tissue was collected from as many subjects as possible based on the availability, as determined by the Pathology Core. Samples were anonymized.

The tissue harvesting protocol consisted of opening of the body cavity using standard techniques and sterile sampling of tissues. The postmortem interval between death and autopsy ranged from 24 to 48 h. Omental, pericardial, subcutaneous abdominal, supraclavicular, paravertebral, periadrenal fat, and urinary bladder were collected at 15 autopsies. Tissue was precured in collaboration with the NIH Clinical Center Department of Pathology. At collection, tissues were resected and placed in 15 mL conical tubes containing normal saline. Immediately after arrival in the laboratory, under sterile conditions, samples were cut into smaller pieces and fixed for immunohistochemistry analysis or stored in 1.7 mL cryovials and immediately placed in liquid nitrogen before transferring to − 80 °C for long-term storage. The samples were not destroyed after use, and some portions remain and may be shared with other researchers, depending on institutional regulations, by contacting Aaron Cypess (aaron.cypess@nih.gov).

### UCP1 Immunofluorescence

Formalin-fixed human adipose tissues were paraffin-embedded and sectioned at 5 μm thickness by American Histolabs Inc. (Gaithersburg, MD, USA). We stained one slide per tissue type from one individual (subject A6 = paravertebral; subject A9 = adrenal, subcutaneous) in two sessions, repeated once. Immunofluorescent staining was performed using Zenon Alexa Fluor conjugated antibody kit (Invitrogen Z25351), where slides were incubated with 1 µg of UCP1 primary antibody (Invitrogen PA1-24894) for 1 h at RT, washed with PBS-Tween, and mounted with DAPI for the visualization of nuclei. For UCP1 (stock concentration 1.3 mg/ml;32 kDa) 0.8 µL stock was added per 20 µL buffer = 1:25. AD served as the positive control (secondary antibody only) as it showed high UCP1 expression and areas of brown adipocyte morphology via H&E staining.

Images were obtained on a Zeiss LSM700 confocal microscope at 20 x objective magnification, and three non-overlapping fields of view were imaged for each tissue sample. To account for differences in cell density, we normalized the UCP1 fluorescence intensity relative to DAPI fluorescence within the same sample. To ensure consistency, we repeated immunofluorescence experiments in two separate sessions using secondary antibodies conjugated to different fluorophores (594 nm and 488 nm). The final reported values represent the average of the normalized UCP1 fluorescence intensities from both experimental sessions (*n* = 2 from 1 sample).

For the tyrosine hydroxylase (TH) staining, one slide per tissue type from one individual (subject A9 = adrenal, paravertebral, subcutaneous) was stained using Zenon Alexa Fluor-488 conjugated antibody kit (Invitrogen Z25351). Slides were incubated with 1 µg of Tyrosine Hydroxylase primary antibody (Abcam ab137869) for 1 h at RT, washed with PBS-T, and mounted with DAPI for the visualization of nuclei. For TH (stock concentration 0.6 mg/ml;58 kDA) 1.67 µL stock was added per 20 µL of buffer = 1:12. AD served as the positive control, as it showed high UCP1 expression and areas of brown adipose tissue morphology under H&E staining. Images were obtained on a Zeiss LSM700 confocal microscope at 20 x objective magnification, and four non-overlapping fields of view were imaged for each tissue sample. To account for differences in cell density, we normalized TH fluorescence intensity relative to DAPI fluorescence within the same sample. The final reported values represent the normalized TH fluorescence intensities of one sample from the same individual.

Validation: UCP1 IHC: Anti UCP1 tested for reactivity to human UCP1; recommended for IHC; cited in 35 publications. TH: Anti-Tyrosine hydroxylase tested for reactivity to human TH; recommended for IHC; cited in 100 + publications.

### RNA Extraction and gene expression

Total RNA was extracted using TRIzol (Invitrogen, Thermo Fisher Scientific) following the manufacturer’s protocol. RNA quantity and quality were determined by spectrophotometry (NanoDrop, Thermo Fisher Scientific). A total of 1 μg of total RNA was reverse transcribed using the High-Capacity cDNA Reverse Transcription kit (Applied Biosystems, Thermo Fisher Scientific). Quantitative reverse transcriptase PCR was run in duplicates using SYBR green fluorescent dye (Bio Basic) and quantified in the ABI PRISM 7900HT Sequence Detection System (Applied Biosystems, Thermo Fisher Scientific). Relative mRNA expression was determined by the Δ-Ct method using *ACTB* as an endogenous housekeeping control. All sequences of primers used in this study are provided in Supplementary Data 11. When comparing gene expression between fat depots, the relative mRNA value, normalized to the SC depot, was reported because SC is the largest and most accessible human WAT depot. All gene expression data are expressed on a log10 scale. Data are represented as mean ± SD. A mixed-effects analysis using donor and depot as random effects with covariates age, sex, postmortem interval, BMI, and RIN was used for statistical analysis.

For qPCR experiments, oligonucleotide designs were based on published oligonucleotide sequences^21^. All oligonucleotides are commercially available through synthesis and were obtained from Integrated DNA Technologies, Inc (Coralville, IA). All sequence information is provided in the Methods section of the manuscript.

### Bulk gene expression in tissue

Reasonable amounts of intact RNA are required to analyze the local differences on a molecular level. Therefore, total RNA was extracted using the RNeasy Kit (Qiagen, Germantown, MD) following the manufacturer’s protocol, and RNA quality and integrity were determined on an Agilent TapeStation (Agilent Technologies, Santa Clara, CA). Given the nature of the degraded samples, as expected the overall average RIN for all samples was between 4.7 ± 1.1. To address this issue of degraded RNA and ensure the best possible libraries preparation, we used the Takara SMARTer Stranded Total RNA-Seq Kit v2 – Pico Input Mammalian. Samples were indexed using the Illumina TruSeq Stranded kit. RNA-seq libraries were then subjected to 2 × 100 paired-end sequencing on an Illumina NextSeq 2000. A minimum of 18 M fragments per sample library was obtained.

Raw sequencing reads were processed using the nf-core/rnaseq pipeline (v3.18.0)^32^ with Nextflow^33^ (v24.09.0) and Java 11. Strandedness was set to “auto” during the strandedness prediction step. Ribosomal RNA was removed using the SortMeRNA tool from its GitHub repository^34^. All other steps were executed with the default parameters of the RNA-Seq pipeline. Reads were aligned to the GRCh38/hg38 reference genome using the STAR aligner (v2.7.11b). Gene-level quantification was performed using Salmon (v1.10.1) against GENCODE v48 (Ensembl v115) gene models. Gene expression values were reported in transcripts per million (TPM) units with additional information on gene length, effective gene length, and read counts, which were converted to gene-level raw counts using tximport (v3.19) in R.

### RNA-Seq secondary analysis

Genes were annotated to the respective HGNC symbol using the human genes dataset (GRCh38.p14) from the BioMart Ensembl database (v4.4) using the R package, biomaRt (v2.58.2). Hierarchical clustering analysis of normalized counts was performed using Euclidean distance and complete linkage clustering methods to create the heatmap. This analysis was conducted with the ggplot2, reshape2 (v1.4.4), pheatmap (v1.0.12), and ggrepel (v0.9.5) R packages. Differential gene expression (DGE) analysis was conducted with R (v4.3.1) using DESeq2 (v3.19). Detailed quality control of RNA-Seq data was performed using RNA-SeQC (v1.1.8), RSeQC (v3.0.1), and MultiQC (v1.7). Genes were considered significantly differentially expressed if they had an adjusted *p*-value (FDR) less than 0.05 and an absolute log2 fold change greater than 1.0 (Supplementary Data 12).

DGE analysis was conducted on various sets of genes: the total gene set, without the urinary bladder depot, without immune genes, with only immune genes, with only white adipocyte genes, without white adipocyte genes, with only brown and white adipocyte genes, with only brown adipocyte genes, without outlier donors, with only miRNA genes, and from three different random samples of genes. All gene sets used in this study are provided in the Supplementary Datasheet. Principal Component Analysis (PCA) was visualized using the top 5000 genes with the most variance and the ggplot2 (v3.5.1) and psych (v2.4.6.26) R packages to display points labeled by tissue type and by individual donors.

### Mouse microarray analysis

Microarray data from a study on the effects of a high-fat diet on adipose tissue of mice^17^ were analyzed. Samples were labeled according to tissue type, individual mice, and dietary condition based on the study. DGE analysis was conducted using DESeq2. Principal Component Analysis (PCA) was visualized using the ggplot2 and psych R packages to display points labeled by tissue type and by individual mice.

### Variance-partitioning model

To quantify the relative contributions of donor and depot to gene expression variability, we employed a variance partitioning approach using the variancePartition package (v1.34.0). VST-normalized gene expression data from the top 5000 most variable genes (as determined by principal component analysis) were analyzed. Linear mixed models for the human autopsy and mice data were specified with random effects for donor and depot or tissue and mice, respectively. This model was fitted across all selected genes, allowing for gene-wise estimation of the proportion of variance attributable to each factor. In a separate model, we incorporated additional covariates to the human autopsy model—including age, BMI, post-mortem interval, RIN, and sex—to assess their contributions alongside donor and depot effects. These covariates were included as fixed or random effects as appropriate, and their relative contributions were quantified in the same manner. Results were reported as mean percentages. To compare variance contribution between donors and depots, we analyzed the proportion of variance explained for each of the 5000 genes using a two-sided Mann-Whitney U test. Statistical significance was defined as *p* ≤ 0.05.

### Clustering quantification

Clustering of points in PCA space was quantified using a modified version of the Calinski-Harabasz (CH) index^16^, adapted to evaluate the separability of individual subgroups (e.g., tissue types or individual donors). To assess the clustering tendency of each subgroup, we employed a binary-split approach, and distances were calculated based on their coordinates in the PCA space from PC1 and PC2. For each subgroup, the following was done:

Points within the subgroup were labeled as one cluster. All other points (outside the subgroup) were labeled as a second cluster. The CH index was then computed for this binary split using the calinhara function from the fpc (v2.2-13) package. The CH index measures the ratio of between-cluster dispersion (BCSS) to within-cluster dispersion (WCSS), scaled by the number of data points (N) and clusters (k):

1 $${CH}=\frac{{BCSS}}{{WCSS}}x\frac{N-k}{k-1}$$

In the context of the binary-split approach, where *k* = 2, the formula simplifies to:

2 $${CH}=\frac{{BCSS}}{{WCSS}}x(N-2)$$

BCSS is the sum of squared distances between cluster centroids and the global mean (between-cluster dispersion). WCSS is the sum of squared distances between points and their respective cluster centroids (within-cluster dispersion). N is the total number of data points.

Higher CH index values indicate that the subgroup is both well-separated from the rest of the data and internally compact.

To compare clustering tendencies between donors and depots, we analyzed CH index scores using a two-sided Mann-Whitney U test with an approximation for large sample sizes. Statistical significance was defined as *p* ≤ 0.05.

### Urinary bladder as a control

Unsupervised clustering was performed using k-means to evaluate whether the UB depot forms a distinct cluster separate from other adipose depots. K-means clustering was applied to the first two principal components (PC1 and PC2) with *k* = 3 clusters, using 150 random initializations to ensure robust results. Cluster assignments were visualized using ggplot2, with points colored by cluster membership and labeled by sample name.

To further assess the distinctiveness of the UB depot, we calculated centroids as the mean PC1 and PC2 coordinates for UB and all other fat depots combined. A PCA plot was generated to display confidence ellipses (95% level) for each depot, with centroids for UB and non-UB marked for clarity. Euclidean distances were computed for each point to both the UB centroid and the non-UB centroid, and these distances were compared to quantify the separation between UB and other depots. Specifically, the mean distance of UB points to the non-UB centroid was compared to the mean distance of non-UB points to the UB centroid. Statistical significance of these differences was assessed using a one-sided Mann-Whitney U test, testing the hypothesis that UB points are significantly farther from the non-UB centroid than non-UB points are from the UB centroid. Statistical significance was defined as *p* ≤ 0.05.

### Cell deconvolution

Cellular deconvolution was performed to determine the proportion of different cell types that make up the bulk RNA-seq samples. Purified cell population TPM values for 20 cell types^5^ were sourced to create custom signature matrices. Deconvolution was performed using CIBERSORTx^35^, applying the respective custom signature matrices along with TPM values from the bulk RNA-seq autopsy data as input. “B-mode” batch correction was applied to the job to handle the technical variations between the cell populations and different experimental conditions^36^. To increase the robustness of the analysis and reduce the noise from the data, we also performed the job using 100 permutations. All analyzed samples had a *P* < 0.0001 and RMSE < 1. We mapped all gene identifiers as well as microarray probe names of AT21 to HGNC (HUGO Gene Nomenclature Committee) IDs using the biomaRt R package. Statistical testing was performed using one-way ANOVA to compare the mean proportion of cell types among the depots. The data are reported as the mean percentage of cells within a given depot ± standard deviation.

### Pathway analysis

Analysis of enriched pathways in adipocyte markers was performed using clusterProfiler (v3.14.3). Adipocyte cluster markers were filtered to a False Discovery Rate adjusted *P* < 0.05, then evaluated for enrichment in GO biological pathways^37^ containing under 300 genes.

### Random forest predictions

To identify a set of highly informative genes for classification, we performed feature selection on the gene expression data. We used the Seurat package (v5.2.0) to identify the most variable genes across all samples. Using the FindVariableFeatures function with the vst selection method, we identified the top 7000 variable genes.

To classify donor identity based on gene expression profiles, we implemented a Random Forest classifier using the randomForest R package (v4.7−1.1). One model was trained on the gene expression data from SC samples, with the donor (or mouse) ID serving as the classification label. The second model was trained on the gene expression data from all samples from Donor 9, with the tissue depot ID serving as the classification label. A forest of 1000 trees was grown (ntree = 1000), and variable importance was assessed to identify the most predictive genes.

To rigorously validate our model, we performed a series of validation tests. First, we conducted a leave-one-donor-out cross-validation (LODO-CV) to assess the model’s ability to generalize to new individuals. This analysis yielded an Area Under the ROC Curve (AUC) of 0.733 with a 95% confidence interval of [0.415-0.963], demonstrating a fair predictive ability that is better than random chance (Supplementary Fig. 12).

To confirm the specificity of this result, we ran a permutation control where donor labels were randomized prior to training in each LODO-CV fold. This control model performed no better than random, with an AUC = 0.493, confirming that our model is learning a true biological signal rather than an artifact of the data structure.

### PET/CT

PET/CT images were acquired and analyzed following previously described methods^13,29,38^. Briefly, PET images from 14 female health volunteers were reconstructed into voxels of 1.45 × 1.45 × 1.5 mm, and CT images were reconstructed into voxels of 0.98 × 0.98 × 1.5 mm. These images were then processed using ImageJ^39^ (NIH, Bethesda, MD). The PET/CT Viewer plug-in, customized for BAT quantification, was utilized in subsequent analyses. Fat was identified using specific CT density ranges (– 300 to – 10 HU) to distinguish it from air and other tissues. The ^18^F-FDG uptake in each PET image voxel was quantified as a standardized uptake value (SUV), normalized to the individual’s lean body mass. Both PET SUV and CT HU criteria were applied to identify metabolically active adipose tissue.

### Statistics and reproducibility

Cellular Deconvolution (CIBERSORTx and the BATLAS algorithm): single factor, one-way ANOVA; to increase the robustness of the analysis and reduce the noise from the data, the processing job was also performed using 100 permutations. All analyzed samples had a *P* < 0.0001 and RMSE < 1.

Quantification of proximity of PCA cluster points: Calinksi-Harabasz Index

Variance-partitioning analysis: two-sided Mann Whitney-U test

Euclidean distance analysis: one-sided Mann Whitney-U test

Random Forest Predictions: To rigorously validate our model, we performed a series of validation tests. First, we conducted a leave-one-donor-out cross-validation (LODO-CV) to assess the model’s ability to generalize to new individuals. This analysis yielded an Area Under the ROC Curve (AUC) of 0.733 with a 95% confidence interval of [0.415–0.963], demonstrating a fair predictive ability that is better than random chance. To confirm the specificity of this result, we ran a permutation control where donor labels were randomized prior to training in each LODO-CV fold. This control model performed no better than random, with an AUC = 0.493, confirming that our model is learning a true biological signal rather than an artifact of the data structure.

To ensure the reproducibility and robustness of our experimental findings, we implemented multiple technical replicates, computational validation steps, and cross-validation techniques throughout the study. Steps to ensure replication include the following:

Immunofluorescence Replicates: To ensure consistency in our imaging, we repeated immunofluorescence experiments in two separate sessions. During these sessions, we used secondary antibodies conjugated to different fluorophores (594 nm and 488 nm).

qPCR Duplicates: All quantitative reverse transcriptase PCR experiments were run in duplicates to ensure technical reproducibility.

Transcriptomic Robustness Checks: To confirm the consistency of our primary transcriptomic finding (that intrasubject variability is lower than intradepot variability), we re-examined the variability using 11 different gene subsets, including random gene subsets. The statistical pattern remained consistently significant across all subsets tested.

Computational Permutations and Initializations: For the cellular deconvolution analysis, the algorithm was performed using 100 permutations to increase robustness and reduce data noise.

For the unsupervised k-means clustering, we utilized 150 random initializations to ensure robust and reproducible cluster assignments.

Predictive Model Validation: To validate our Random Forest model, we performed a series of validation tests.

We conducted a LODO-CV to verify the model’s ability to generalize to new individuals.

We confirmed the specificity of the biological signal by running a permutation control where donor labels were randomized prior to training, which expectedly resulted in random performance.

We trained the model on subcutaneous samples and successfully tested it on withheld visceral (omental) samples, achieving 80% accuracy.

For the autopsy part of the study, traditional blinding to group allocation was not applicable as it was an observational, comparative study characterizing inherent biological properties (anatomical adipose depots and individual donor identities). However, to prevent investigator bias during data collection and analysis, we implemented the following:

Standardized Transcriptomic Pipelines: The processing of raw RNA-seq data was fully automated using the standardized nf-core/rnaseq pipeline, STAR aligner and Salmon for objective gene-level quantification.

Unsupervised Analyses: Exploratory analyses were completely unsupervised. For example, the k-means clustering used to evaluate the UB control used an algorithm to group the samples purely based on data patterns without any prior knowledge of the sample labels from the investigators.

Permutation Controls: To validate our Random Forest predictive models, we implemented a permutation control where donor labels were randomized. This ensured the model learned a true biological signal rather than reflecting artifacts or structural biases.

For the prospective clinical trial, the sample size was based on log_10_BAT activity. We determined that a sample size of 14 women was necessary to detect whether a change in BAT metabolic activity was different from a null hypothesis of 0.00 with 80% power using a paired Student’s *t* test with a significance level of 0.05 and a correlation (*R*) of 0.50. Treatment was not blinded for both the staff and study participants. The experiments were not randomized. No data were excluded.

Objective Imaging Thresholds: For the 18F-FDG PET/CT analysis, adipose tissue was not identified by investigators. Instead, it was objectively defined using predefined CT density ranges (− 300 to − 10 HU) combined with a customized PET/CT Viewer plug-in to extract standardized uptake values (SUV).

### Reporting summary

Further information on research design is available in the Nature Portfolio Reporting Summary linked to this article.

## Supplementary information


Supplementary Information
Description of Additional Supplementary Files
Supplementary Data 1
Supplementary Data 2
Supplementary Data 3
Supplementary Data 4
Supplementary Data 5
Supplementary Data 6
Supplementary Data 7
Supplementary Data 8
Supplementary Data 9
Supplementary Data 10
Supplementary Data 11
Supplementary Data 12
Reporting Summary
Transparent Peer Review file


## Source data


Source Data


## Data Availability

The RNA-seq data generated in this study have been deposited in the NCBI GEO database under accession code GSE334145. The source data generated in this study underlying Figs. 1c, d, f–h, 2a–d, 3a, b, 4b, and Supplementary Figs. 1–5 are provided in the Source Data and Supplementary Data files. Source data are provided in this paper.

## Code availability

All analyses related to RNA-seq processing, statistical analyses, cell deconvolution, the modified Calinski–Harabasz algorithm, pathway analysis, and random forest predictions were done using publicly available codes.
