# Supplementary material for: Transcriptomic profiling of human brown and white adipose tissue depots
Source: Nat Commun. 2026 Jul 29;17:9181. doi: 10.1038/s41467-026-76065-7 (PMC13522497; doi:10.1038/s41467-026-76065-7)
Supplement: Supplementary file 1 [file 41467_2026_76065_MOESM15_ESM.pdf]

nature portfolio

Corresponding author(s): Aaron M. Cypess

Last updated by author(s): Jun 3, 2026

Reporting Summary

Nature Portfolio wishes to improve the reproducibility of the work that we publish. This form provides structure for consistency and transparency in reporting. For further information on Nature Portfolio policies, see our Editorial Policies and the Editorial Policy Checklist.

Statistics

For all statistical analyses, confirm that the following items are present in the figure legend, table legend, main text, or Methods section.

n/a	Confirmed	
☐	☒	The exact sample size (*n*) for each experimental group/condition, given as a discrete number and unit of measurement
☐	☒	A statement on whether measurements were taken from distinct samples or whether the same sample was measured repeatedly
☐	☒	The statistical test(s) used AND whether they are one- or two-sided<br>*Only common tests should be described solely by name; describe more complex techniques in the Methods section.*
☐	☒	A description of all covariates tested
☐	☒	A description of any assumptions or corrections, such as tests of normality and adjustment for multiple comparisons
☐	☒	A full description of the statistical parameters including central tendency (e.g. means) or other basic estimates (e.g. regression coefficient) AND variation (e.g. standard deviation) or associated estimates of uncertainty (e.g. confidence intervals)
☐	☒	For null hypothesis testing, the test statistic (e.g. *F*, *t*, *r*) with confidence intervals, effect sizes, degrees of freedom and *P* value noted<br>*Give P values as exact values whenever suitable.*
☒	☐	For Bayesian analysis, information on the choice of priors and Markov chain Monte Carlo settings
☒	☐	For hierarchical and complex designs, identification of the appropriate level for tests and full reporting of outcomes
☒	☐	Estimates of effect sizes (e.g. Cohen's *d*, Pearson's *r*), indicating how they were calculated

Our web collection on statistics for biologists contains articles on many of the points above.

Software and code

Policy information about availability of computer code

Data collection	No software was used to collect the human autopsy data. For the mouse microarray data, expression data from GEO Series GSE15822 was downloaded from the Gene Expression Omnibus (GEO) website and analyzed locally.<br>-UCP1: 2D IHC Images acquired on LSM700 (Zeiss) confocal microscope<br>-TH: 2D IHC Images acquired on LSM700 (Zeiss) confocal microscope<br>-Bulk RNAseq: NGS raw data collected by MedGenome (Signios Bio, CA)<br>-PET/CT: Subjects were dosed with 10 mCi of 18F-FDG by i.v. injection 60–90 min before scan acquisition using a Siemens Biograph mCT (Siemens Healthcare)
Data analysis	qPCR data were analyzed using GraphPad Prism (Version 10.3.0). RNA-seq data preprocessing was performed with the nf-core/rnaseq pipeline (v3.18.0) using Nextflow (v24.09.0) and Java 11. Secondary analysis of RNA-seq data utilized the BioMart Ensembl database (v4.4) via the R package biomaRt (v2.58.2). Additional R packages used for analysis and visualization included DESeq2 (v3.19), ggplot2 (v3.5.1), psych (v2.4.6.26), geepack (v1.3.12), Seurat (v5.1.0), randomForest (v4.7-1.1), and fpc (v2.2-13). Deconvolution was performed using CIBERSORTx (v1)<br><br>-Image handling was performed using ImageJ(v1.54)<br>-RNAseq data was processed using nf-core/rnaseq pipeline (v3.18.0) with Nextflow (v24.09.0) and Java (v11). Ribosomal RNA was removed using SortMeRNA (v4.3.7). Reads were aligned to the GRCh38/hg38 reference genome using STAR aligner (v2.7.11b) and processed using Trimgalore (v0.6.7) and cutadapt (v3.4). Quality control of RNA-Seq data was performed using RNA-SeQC (v1.1.8), RSeQC (v3.0.1), and MultiQC (v1.7).<br>Gene-level quantification was performed using Salmon (v1.10.1) against GENCODE v48 (Ensembl v115) gene models. Gene-level raw counts

were generated using tximport (v3.19).
-Data was visualized with ggplot, reshape2 (v1.4.4), pheatmap (v1.0.12), ggrepel (v0.9.5), and psych (v2.4.6.26) using R (v4.3.1)
-Differential gene expression analysis was achieved using DESeq2 (v3.19)
-Variance partitioning models were generated using variancePartition package (v1.34.0)
-Clustering quantification was achieved using Calinski-Harabasz index with the calinhara function from fpc (v2.2-13)
-Cell deconvolution was performed using CIBERSORTx
-Pathway analysis was performed using clusterProfiler (v.3.14.3)
-Random forest predictions were performed using randomForest (v4.7-1.1) on most variable genes identified by FindVariableFeatures function of Seurat (v5.2.0)
-PET/CT volumes were processed using ImageJ and PET/CT viewer plug-in customized for BAT quantification

For manuscripts utilizing custom algorithms or software that are central to the research but not yet described in published literature, software must be made available to editors and reviewers. We strongly encourage code deposition in a community repository (e.g. GitHub). See the Nature Portfolio guidelines for submitting code & software for further information.

Data

Policy information about availability of data

All manuscripts must include a data availability statement. This statement should provide the following information, where applicable:

- Accession codes, unique identifiers, or web links for publicly available datasets
- A description of any restrictions on data availability
- For clinical datasets or third party data, please ensure that the statement adheres to our policy

The original data related to the tables and figures can be found in this repository: https://osf.io/zbu64.

Research involving human participants, their data, or biological material

Policy information about studies with human participants or human data. See also policy information about sex, gender (identity/presentation), and sexual orientation and race, ethnicity and racism.

Reporting on sex and gender	There is one prospective clinical trial described. Subjects are described as female, referring first to their gender identification and then by their biological status as determined through imaging and identification of a uterus.<br>For the autopsies, gender was not known to us, only the reported sex of the deceased subjects.
Reporting on race, ethnicity, or other socially relevant groupings	Race and ethnicity were not reported.
Population characteristics	For the autopsy subjects, age was the relevant co-variate that was analyzed. We report that donors within 0.5 standard deviations from the mean age range exhibited the least variability, clustering most tightly
Recruitment	Healthy volunteers were recruited by word of mouth or through the Patient Recruitment and Public Liaison Office of the Clinical Center and provided written informed consent according to Declaration of Helsinki principles. Compensation was provided.
Ethics oversight	This study was approved by the Human Studies Institutional Review Board of the NIDDK and the NIH Radiation Safety Committee.

Note that full information on the approval of the study protocol must also be provided in the manuscript.

Field-specific reporting

Please select the one below that is the best fit for your research. If you are not sure, read the appropriate sections before making your selection.

[x] Life sciences   [ ] Behavioural & social sciences   [ ] Ecological, evolutionary & environmental sciences

For a reference copy of the document with all sections, see nature.com/documents/nr-reporting-summary-flat.pdf

Life sciences study design

All studies must disclose on these points even when the disclosure is negative.

Sample size	For the clinical trial, the sample size was determined as described in the original publication (PMID 31961826). There we reported the following approach to the sample size calculation:<br>Our previous studies showed that BAT activity is not normally distributed; rather, it is right skewed (7, 22). Thus, our sample-size determination was based on log10BAT activity. Since there were no published studies assessing the effects of mirabegron on log10BAT activity, we studied a similar cohort exposed to chronic cold exposure (32) and found changes in log10 BAT volume of +0.21 ± 0.25 mL. Since BAT volume correlates with BAT activity (12), we then determined that a sample size of 14 women was necessary to detect whether a change in BAT metabolic activity was different from a null hypothesis of 0.00 with 80% power using a paired Student's t test with a significance level of 0.05 and a correlation (R) of 0.50. Treatment was non-blinded for both the staff and study participants.<br><br>For the autopsy study, we collected tissue from as many volunteers as we were given access to by the Anatomic Pathology group.

Data exclusions	The tissue samples—including up to six types of adipose tissue and the urinary bladder—were collected from 15 autopsies. Prior to RNA sequencing, samples from four donors were excluded based on predefined criteria: either the absence of urinary bladder tissue or the absence of all adipose tissue types of interest.
Replication	To further explore the robustness of our initial findings, we re-examined the variability using multiple different gene subsets. The results are in Supplementary Fig. 2a-c and Supplementary Table 4. These analyses confirmed that the general trend holds across a variety gene sets: intradepot variability was greater than intrasubject variability.
Randomization	For the clinical trial, there were no components that required randomization. Each subject took mirabegron for 4 weeks.
Blinding	Treatment was nonblinded for both the staff and study participants.

Reporting for specific materials, systems and methods

We require information from authors about some types of materials, experimental systems and methods used in many studies. Here, indicate whether each material, system or method listed is relevant to your study. If you are not sure if a list item applies to your research, read the appropriate section before selecting a response.

Materials & experimental systems

n/a	Involved in the study
☐	☒ Antibodies
☒	☐ Eukaryotic cell lines
☒	☐ Palaeontology and archaeology
☒	☐ Animals and other organisms
☐	☒ Clinical data
☒	☐ Dual use research of concern
☒	☐ Plants

Methods

n/a	Involved in the study
☒	☐ ChIP-seq
☒	☐ Flow cytometry
☒	☐ MRI-based neuroimaging

Antibodies

Antibodies used	-UCP1 IHC: Anti UCP1 polyclonal antibody (Thermo Fisher/Invitrogen PA1-24894) followed by Zenon Alexa Fluor conjugated secondary antibody (Thermo Fisher/Invitrogen Z25351)<br>-TH: Anti Tyrosine hydroxylase (Abcam ab137869)) followed by Zenon Alexa Fluor conjugated secondary antibody (Thermo Fisher/ Invitrogen Z25351)
Validation	Anti UCP1 tested for reactivity to human UCP1; recommended for IHC; cited in 35 publications. TH: Anti Tyrosine hydroxylase tested for reactivity to human TH; recommended for IHC; cited in 100+ publications.

Clinical data

Policy information about clinical studies

All manuscripts should comply with the ICMJE guidelines for publication of clinical research and a completed CONSORT checklist must be included with all submissions.

Clinical trial registration	NCT03049462
Study protocol	https://clinicaltrials.gov/ct2/show/NCT03049462
Data collection	Data collection was done at the NIH Clinical Center in Bethesda, MD, which was the single site where the clinical trial was conducted.
Outcomes	The primary endpoint was the change in BAT metabolic activity as measured by [18F]-2-fluoro-d-2-deoxy-d-glucose (18F-FDG) PET/ CT. Secondary endpoints included resting energy expenditure (REE), plasma metabolites, and glucose and insulin metabolism as assessed by a frequently sampled intravenous glucose tolerance test.

Plants

Seed stocks	n/a
Novel plant genotypes	n/a
Authentication	n/a
