# Supplementary material for: Transcriptomic profiling of human brown and white adipose tissue depots
Source: Nat Commun. 2026 Jul 29;17:9181. doi: 10.1038/s41467-026-76065-7 (PMC13522497; doi:10.1038/s41467-026-76065-7)
Supplement: Supplementary file 2 [file 41467_2026_76065_MOESM16_ESM.pdf]

nature portfolio

Peer Review File

Transcriptomic profiling of human brown and white adipose tissue depots

Corresponding Author: Dr Aaron Cypess

This manuscript has been previously reviewed at another journal. This document only contains information relating to versions considered at Nature Communications.

This file contains all reviewer reports in order by version, followed by all author rebuttals in order by version.

Version 1:

Reviewer comments:

Reviewer #1

(Remarks to the Author)
The authors have adequately address my questions, congratulations to their work.

Reviewer #2

(Remarks to the Author)
I thank the authors for their efforts in addressing the reviewers' comments. I particularly acknowledge the improved reporting of quality control parameters and the additional analyses that were performed in response to the first round of review.
That said, I believe there are still important points that should be addressed before the manuscript can be considered fully satisfactory.
Explicit discussion of limitations: While the authors provided detailed and thoughtful responses to the reviewers, many of the corresponding changes in the manuscript itself remain largely cosmetic. In particular, the limitations of the study, and of several applied analytical approaches, are not clearly or explicitly discussed in the main text. Given the substantial heterogeneity of the human cohort, the post-mortem nature of the samples, and the exploratory use of several analytical tools (e.g., deconvolution), a more transparent and critical discussion of these limitations is warranted to appropriately frame the conclusions.
Figure readability and visual consistency: Several display items remain difficult to interpret. For example, in Fig. 1c, the UpSet plot is an improvement over the previous Venn diagram; however, the numbers above the bars are not legible and may not be necessary. In addition, filtering or grouping intersections could further improve clarity. Similarly, numeric labels in Fig. 1f and 1h are difficult to read.
More generally, figure panels use a variety of font sizes, stylings, and color schemes. In some cases, the same depots are represented with different colors across panels (e.g., between Fig. 1a and Fig. 1e), which complicates interpretation. Greater visual consistency across figures would substantially improve readability.
Interpretation of mouse–human differences: The authors state that "genetics, diet, and disease play impactful roles" in regulating adipose tissue depot profiles. While this is certainly true, it would be helpful if the authors could also speculate, at least briefly, on what might drive the relative lack of such variability in the mouse data compared to the human samples. Explicitly addressing why these factors manifest so differently across species would help contextualize the comparison.
This point also relates to my main remaining concern. While I appreciate the additional analyses excluding female donors and donors without malignancies, the fundamental asymmetry between the datasets remains: the mouse data derive from an inbred strain with minimal expected inter-individual variability, whereas the human cohort is genetically and clinically heterogeneous. In this context, many of the observed differences between depot-driven and donor-driven clustering are, in my opinion, overinterpreted.
Given that large inter-individual variation is not expected in an inbred mouse line, it remains unclear what biological insight the authors aimed to gain by directly contrasting this model with a heterogeneous human cohort, particularly when the central conclusion hinges on differences in clustering structure. A clearer justification of this comparison, and a more cautious interpretation of the resulting differences, would strengthen the manuscript.

Reviewer #3

(Remarks to the Author)
I have carefully reviewed the revised version of the manuscript and the authors' detailed point-by-point responses. The authors have addressed all of my previous comments and concerns thoroughly and thoughtfully.

The revisions have substantially improved the clarity, rigor, and overall quality of the manuscript. I have no further comments or requests for revision at this stage.

I believe the manuscript is now suitable for publication in Nature Communications, and I recommend acceptance.

Version 2:

Reviewer comments:

Reviewer #2

(Remarks to the Author)
Thank you for taking the time to address my previous comments. I appreciate the revisions, improvements to the figures and the clarifications provided. All of my points have been adequately addressed, and I have no further concerns at this stage.

Open Access This Peer Review File is licensed under a Creative Commons Attribution 4.0 International License, which permits use, sharing, adaptation, distribution and reproduction in any medium or format, as long as you give appropriate credit to the original author(s) and the source, provide a link to the Creative Commons license, and indicate if changes were made.
In cases where reviewers are anonymous, credit should be given to 'Anonymous Referee' and the source.
The images or other third party material in this Peer Review File are included in the article's Creative Commons license, unless indicated otherwise in a credit line to the material. If material is not included in the article's Creative Commons license and your intended use is not permitted by statutory regulation or exceeds the permitted use, you will need to obtain permission directly from the copyright holder.
To view a copy of this license, visit https://creativecommons.org/licenses/by/4.0/

Response NCOMMS-25-46998B

We would like to thank the reviewers once again for their careful and detailed analysis of our manuscript. We have now addressed the newest set of comments and believe the revision is further improved.

The following is a point-by-point response to the reviewer’s comments and a description of how and where in the text the modifications were made. The reviewer’s comments are in bold, and our responses begin with “>” and are in regular font. The revised portions of the text are in the marked copy and indicated via “blue” font, with underlining to show additions and strike-through to show deletion. The lines where the changes were made are highlighted in this document and refer to the clean version of the revised manuscript.

Reviewers' comments:

Reviewer #2 (Remarks to the Author):

I thank the authors for their efforts in addressing the reviewers’ comments. I particularly acknowledge the improved reporting of quality control parameters and the additional analyses that were performed in response to the first round of review.
That said, I believe there are still important points that should be addressed before the manuscript can be considered fully satisfactory.

A. Explicit discussion of limitations: While the authors provided detailed and thoughtful responses to the reviewers, many of the corresponding changes in the manuscript itself remain largely cosmetic. In particular, the limitations of the study, and of several applied analytical approaches, are not clearly or explicitly discussed in the main text. Given the substantial heterogeneity of the human cohort, the post-mortem nature of the samples, and the exploratory use of several analytical tools (e.g., deconvolution), a more transparent and critical discussion of these limitations is warranted to appropriately frame the conclusions.

>We appreciate the reviewer’s concern about the insufficient discussion of limitations related to the study itself and the analytical approaches. We have now revised the relevant sections so that there is a larger, more transparent, and critical discussion of the limitations (lines 236-250):

“This study is limited by several factors. The heterogeneity of the human cohort may have led to an identification of correlations that were specific to the distinct group while at the same time missed physiological patterns that would have been found within a larger cohort. The use of autopsy tissue may have brought variations in post-mortem interval and differential degradation of RNA, which could have affected the transcriptomic profiles. Therefore, the small sample size and specific population reduce the generalizability of the findings. On the analytical side, several exploratory tools were used, such as cellular deconvolution, the modified CH-Index, and Random Forest classification. These carry the risk of bias and limited generalizability. For

example, cell deconvolution of bulk RNA-seq data can be impacted by the accuracy and generality of cell signature assignments offered by the applied reference data. As this field rapidly evolves and single-cell human tissue atlases continue to grow, we anticipate that comprehensive adipose tissue references and improved algorithms for bulk RNA-seq deconvolution will become available. Larger and functional studies are needed to directly test the physiological relevance of the transcriptomic profiling reported here."

B. Figure readability and visual consistency: Several display items remain difficult to interpret. For example, in Fig. 1c, the UpSet plot is an improvement over the previous Venn diagram; however, the numbers above the bars are not legible and may not be necessary. In addition, filtering or grouping intersections could further improve clarity. Similarly, numeric labels in Fig. 1f and 1h are difficult to read.

>We acknowledge the concerns about readability and visual consistency. We have modified the UpSet plot in Fig. 1c such that the numbers above the bars have been removed. We have filtered and grouped the intersections to further improve clarity.
The numeric labels in Fig. 1f and 1h have been removed and are in the "Deconvolution StatTest" worksheet of the Extended Data Sheet file for readers who would like to see the original values.

C. More generally, figure panels use a variety of font sizes, stylings, and color schemes. In some cases, the same depots are represented with different colors across panels (e.g., between Fig. 1a and Fig. 1e), which complicates interpretation. Greater visual consistency across figures would substantially improve readability.

>We have harmonized the color scheme across panels so that they are consistent with the reference Fig. 1a. This can be seen in the revised Fig. 1e, which is now consistent with Fig. 1a and Fig. 2h.

D. Interpretation of mouse–human differences: The authors state that "genetics, diet, and disease play impactful roles" in regulating adipose tissue depot profiles. While this is certainly true, it would be helpful if the authors could also speculate, at least briefly, on what might drive the relative lack of such variability in the mouse data compared to the human samples. Explicitly addressing why these factors manifest so differently across species would help contextualize the comparison.

>We appreciate the reviewer's concerns about the current approach to the mouse-human differences. The goal of this manuscript is to communicate the distinct characteristics of the human BAT and WAT depots more than to highlight the differences in adipose tissue transcriptomics between mice and humans. We have speculated briefly on what might drive the relative lack of such variability in the mouse data compared to the human samples, which can be found in the text (lines 210-215).

"The comparatively lower transcriptomic variability in the mouse dataset compared to the humans studied here could arise from several drivers leading to essential differences in the datasets. The mice were genetically identical; had the same environments in terms of housing and temperature; consumed one of two defined diets; lived the same number of weeks; and remained otherwise healthy. These features combined will minimize inter-individual variability and have been shown to affect mouse transcriptomics[28]."

E. This point also relates to my main remaining concern. While I appreciate the additional analyses excluding female donors and donors without malignancies, the fundamental asymmetry between the datasets remains: the mouse data derive from an inbred strain with minimal expected inter-individual variability, whereas the human cohort is genetically and clinically heterogeneous. In this context, many of the observed differences between depot-driven and donor-driven clustering are, in my opinion, overinterpreted.
Given that large inter-individual variation is not expected in an inbred mouse line, it remains unclear what biological insight the authors aimed to gain by directly contrasting this model with a heterogeneous human cohort, particularly when the central conclusion hinges on differences in clustering structure. A clearer justification of this comparison, and a more cautious interpretation of the resulting differences, would strengthen the manuscript.

>We acknowledge the reviewer's main remaining concern. We have taken several steps to address them in the revised text. First, in the abstract, we have removed reference to the comparison to mice since the goal is to focus on the findings among the human adipose tissue depots (line 28). We removed the analogous language from the Conclusion (lines 251-252).

We have also written a clearer justification of the comparison with a more cautious interpretation of the resulting differences (lines 210-222).

"The comparatively lower transcriptomic variability in the mouse dataset compared to the humans studied here could arise from several drivers leading to essential differences in the datasets. The mice were genetically identical; had the same environments in terms of housing and temperature; consumed one of two defined diets; lived the same number of weeks when the interventions began; and remained otherwise healthy. These features combined will minimize inter-individual variability and have been shown to affect mouse transcriptomics[28]. Not one of these factors is applicable to the subjects studied here or humans in general, who are genetically, clinically, and environmentally heterogeneous in both known and unknown ways. Nevertheless, a value of this comparison is that inbred mice are often used to understand the behavior of analogous human tissues, so the similarities and differences identified here will be supportive in refining models of adipose tissue physiology. It will be informative to see how variations in these factors in outbred or wild mice affect their adipose tissue transcriptomics when compared to both inbred mice and to humans."
