# Supplementary material for: Transcriptomic profiling of human brown and white adipose tissue depots
Source: Nat Commun. 2026 Jul 29;17:9181. doi: 10.1038/s41467-026-76065-7 (PMC13522497; doi:10.1038/s41467-026-76065-7)
Supplement: Supplementary file 3 [file 41467_2026_76065_MOESM1_ESM.pdf]

Supplementary Figure 1 Immunofluorescence, qPCR analysis, expression matrix of different brown, beige, and white adipocyte-associated genes and the donors' depots.

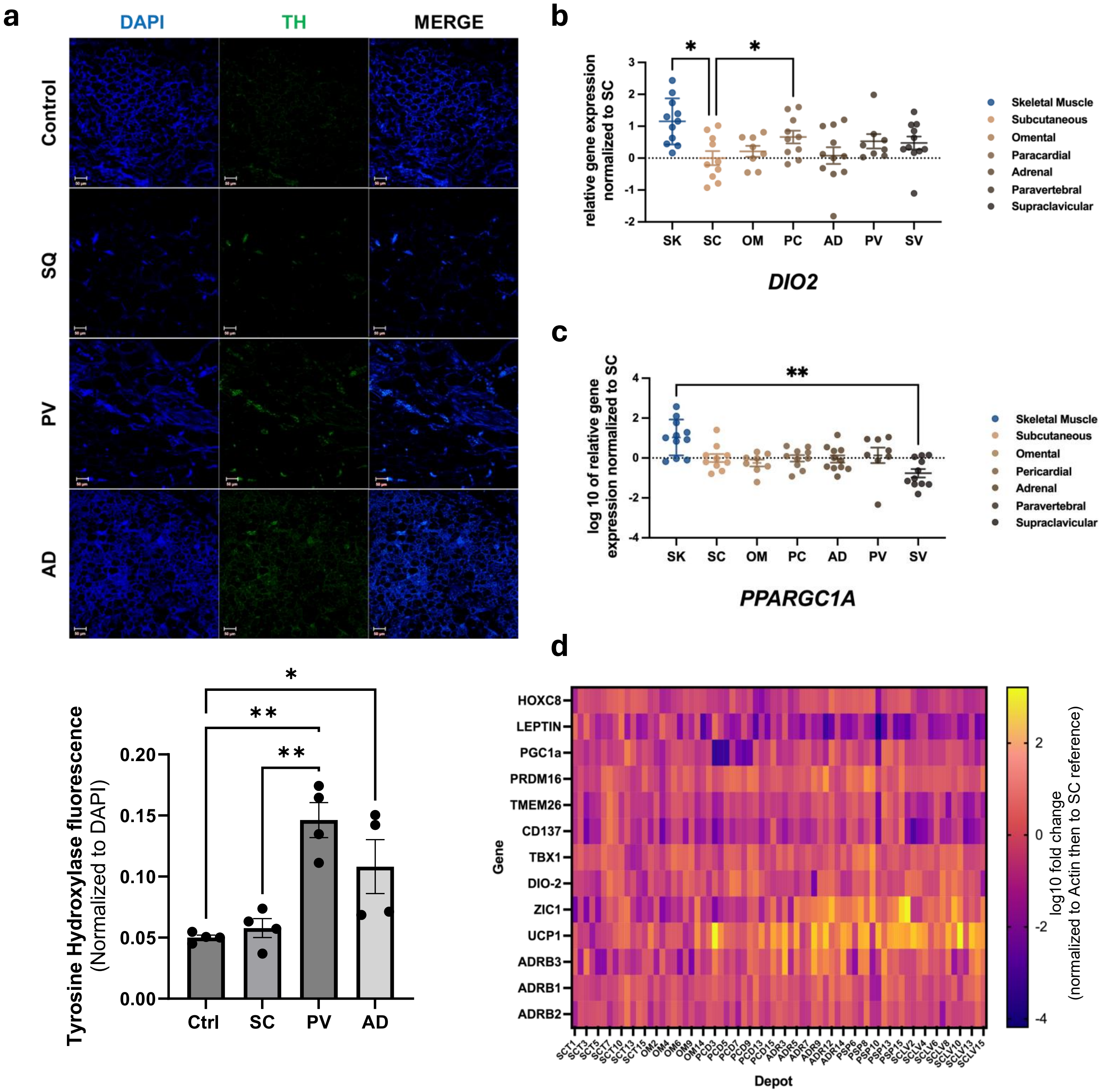

Tyrosine hydroxylase (TH) immunofluorescence in (**a**) SC, PV, and AD depots with the AD depot as a primary antibody control. Scale bar depicts 50 µm. Each bar represents 1 subject (#9); each point represents 1 image. Error bars are SEM of the n=4 points. * $p < 0.05$, ** $p < 0.01$, Tukey's multiple comparison test, two-sided. qPCR analysis of (**b**) *DIO2* and (**c**) *PPARGC1A* (two thermogenic gene markers). Gene expression data were normalized to ACTIN, the SC depot, and then log-transformed and analyzed using a mixed-effects analysis. For both, n = 10 (SK), 10 (SC), 8 (OM), 10 (PC), 8 (PV), 11 (AD), 11 (SV). Specific *p*-values for the comparisons SK vs SC: 0.0108; SC vs PC: 0.0129; SK vs SV: 0.0020 (**d**) expression matrix depicting expression levels of adipocyte-associated genes. The color scale is based on the log10 fold change, normalized to Actin then to reference SC depot.

Supplementary Figure 2 There are transcriptional differences in regulation between all depots relative to the subcutaneous depot.

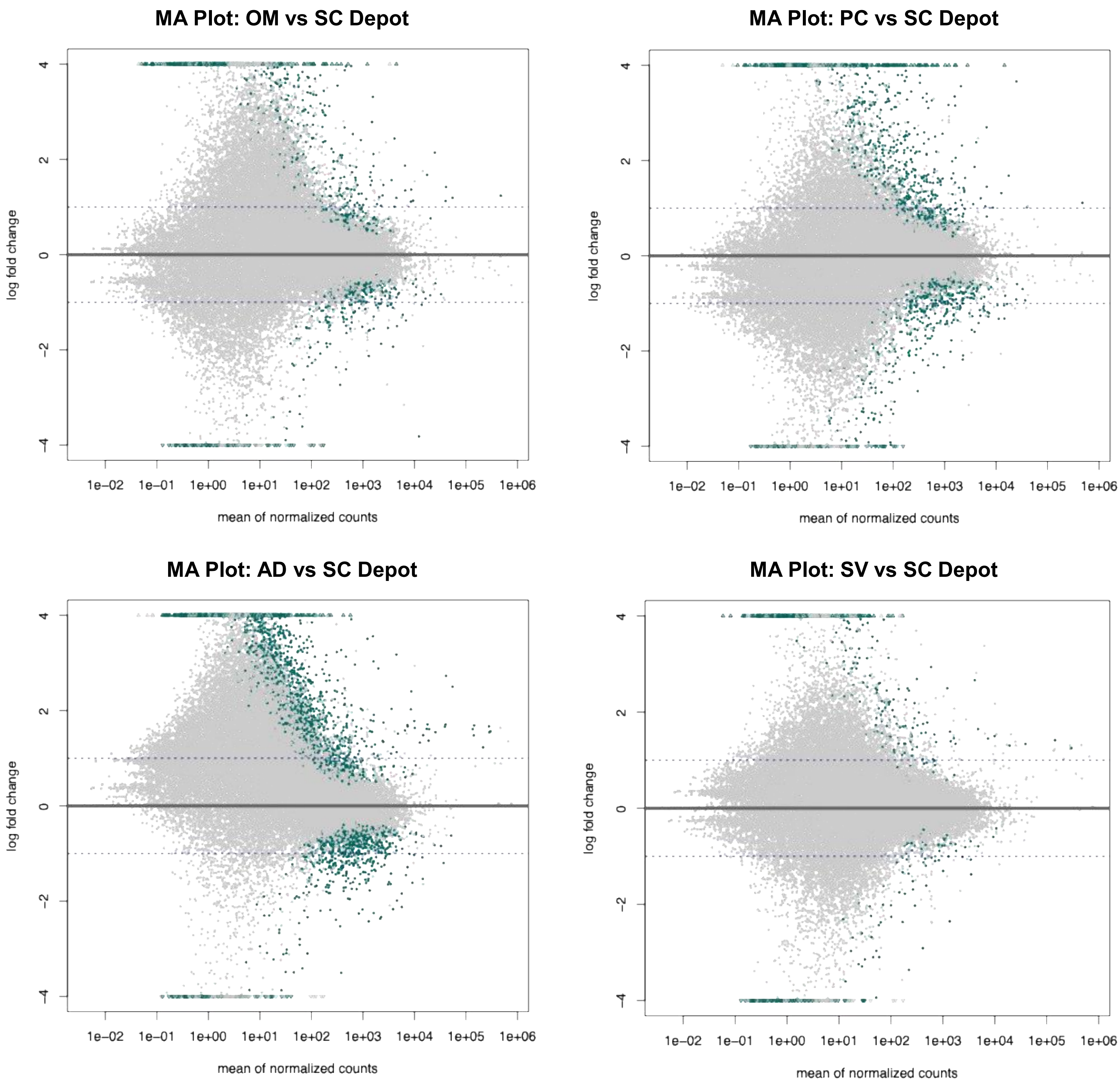

MA plots visualizing the differential expression between subcutaneous (SC) and omental (OM) (**top left**), pericardial (PC) (**top right**), adrenal (AD) (**bottom left**), and supraclavicular (**bottom right**) depots relative to the subcutaneous depot. Genes with a statistically significant adjusted *p*-value (< 0.05) are highlighted in teal.

Supplementary Figure 3 Deconvolution to determine the presence of structural Wnt-regulated adipose tissue-resident (SWAT), adipogenic, and progenitor cell types

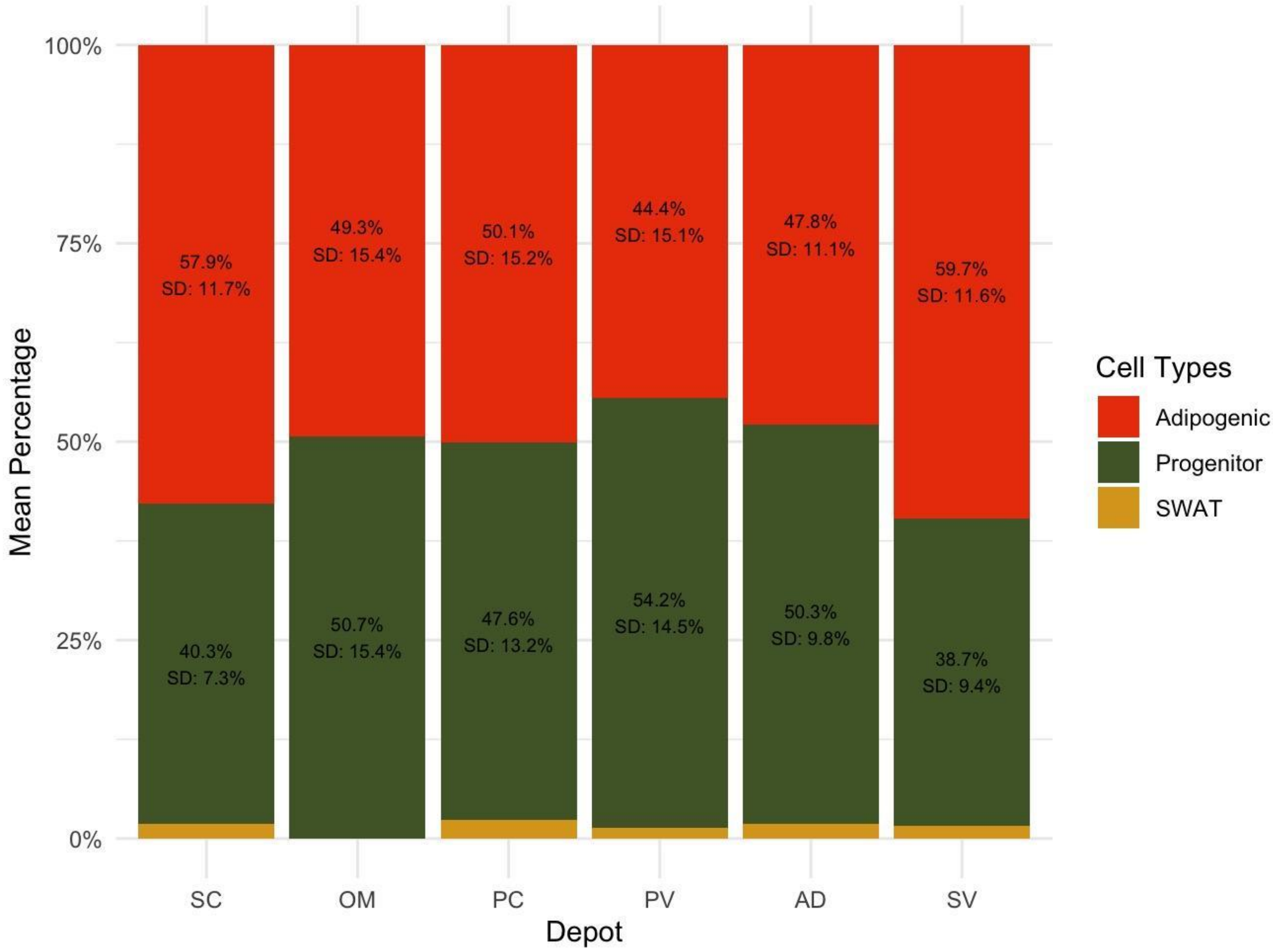

Supplementary Figure 4 All depots have different gene pathways that are enriched or suppressed relative to the subcutaneous depot.

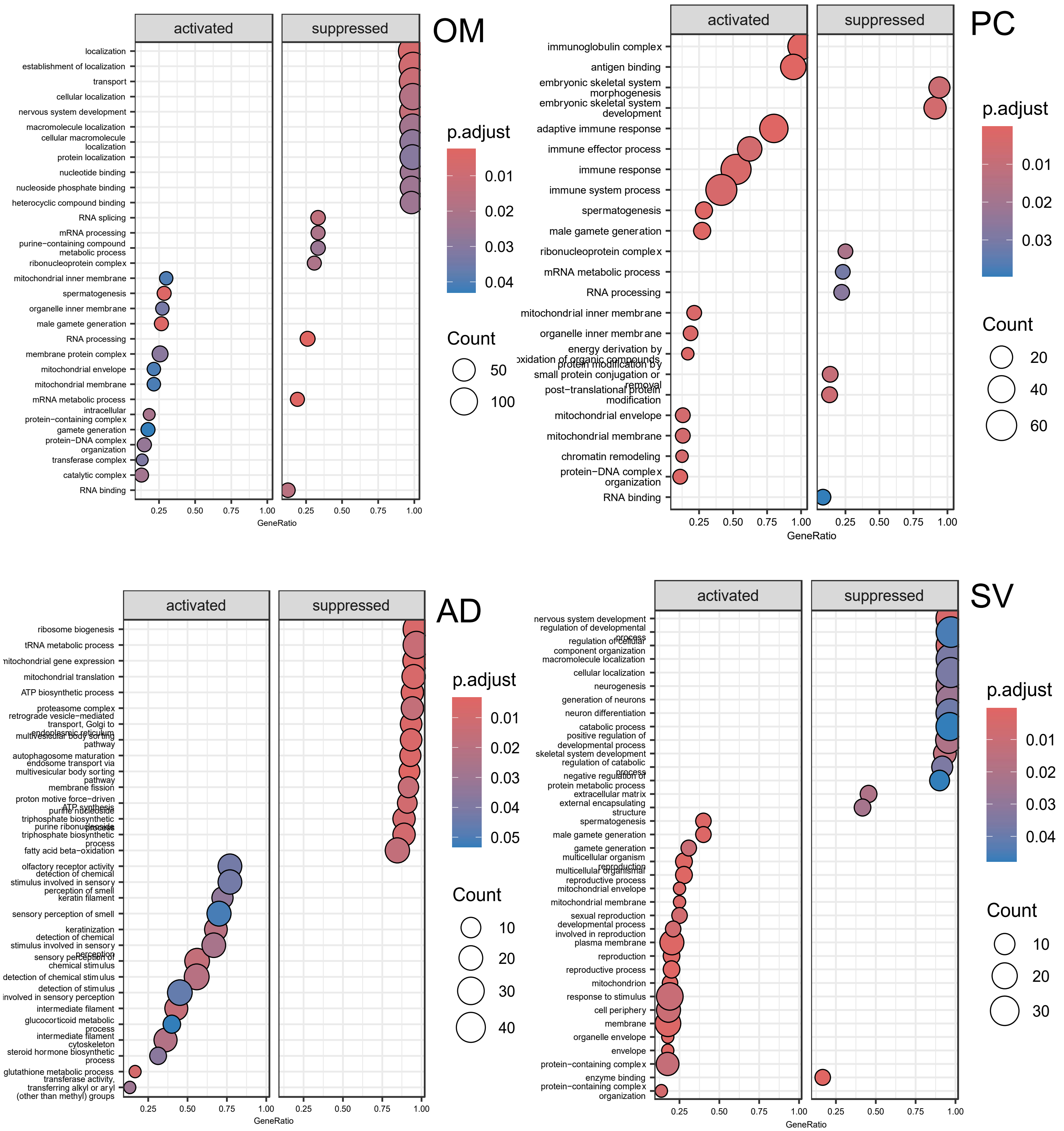

Dot plot analysis showing representative Gene Ontology (GO) pathways enriched in omental (OM) (**top left**), pericardial (PC) (**top right**), adrenal (AD) (**bottom left**), and supraclavicular (SV) (**bottom right**) depots relative to the subcutaneous depot.

Supplementary Figure 5 Variance-partitioning models show that donors contribute the most to the variance of the top 5,000 differentially expressed genes (DEG).

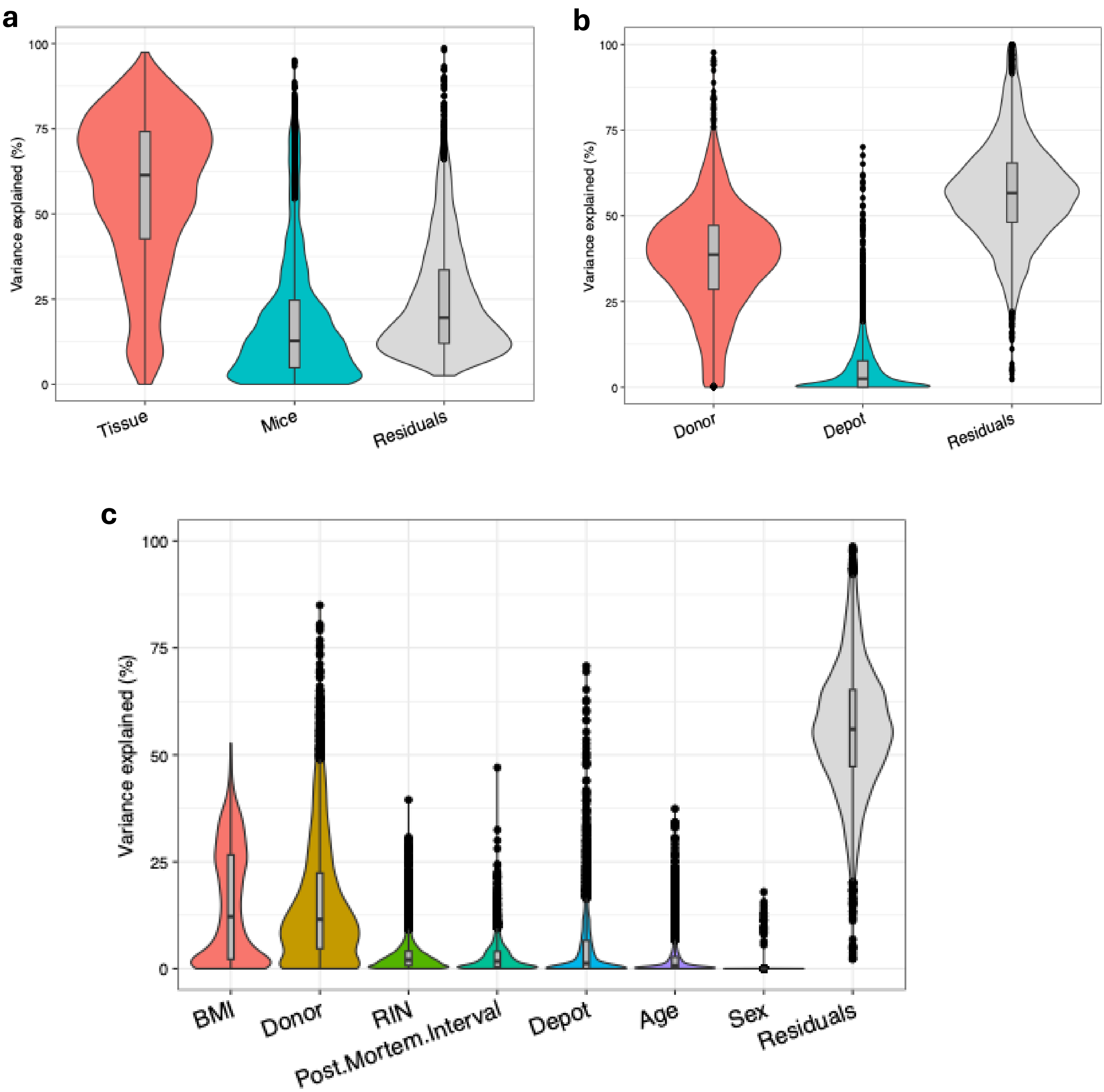

Violin plot of the top 5000 differentially expressed genes showing percentage variance accounted for (**a)** a mouse model with only tissue and donors as random effect variables; (**b)** in a human autopsy model with only donors and depots as random effect variables; and (**c)** in a human autopsy model with donors, depots, and sex as random effect variables and BMI, RIN, post-mortem interval, and age as fixed effect variables. Median is shown by central dot in grey bar indicating interquartile range (25th to 75th percentile); whiskers show min/max values.

Supplementary Figure 6 Donors cluster more closely than depots with different gene combinations.

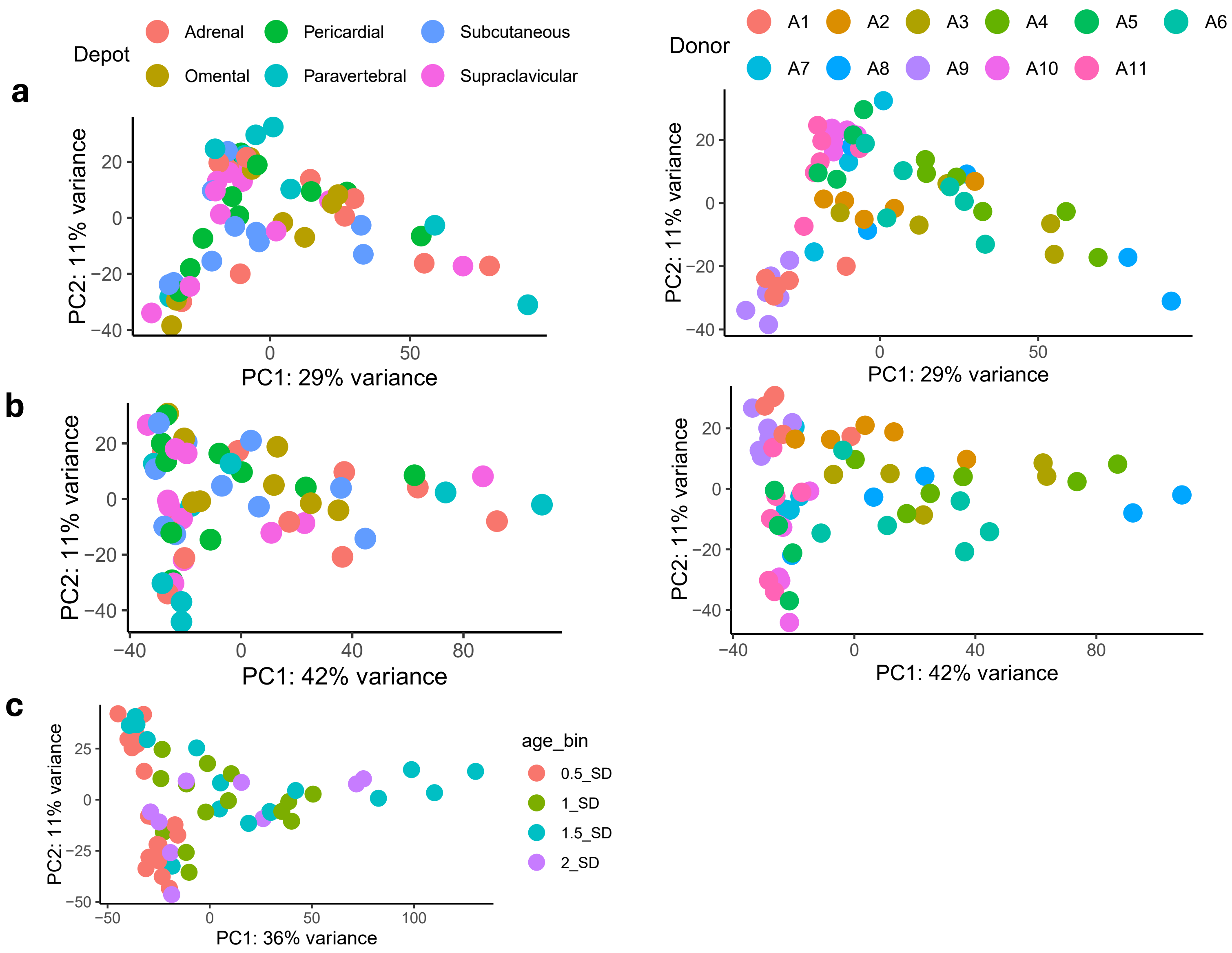

PCA analyses with samples colored by depot (left) and donor (right): (**a**) all adipogenic genes; (**b**) excluding immune function-related genes; (**c**) grouped by donor age bins, where bins represent standard deviations from mean donor age.

Supplementary Figure 7 Donors cluster more closely than depots when the human dataset has only males and malignancy as cause of death.

a

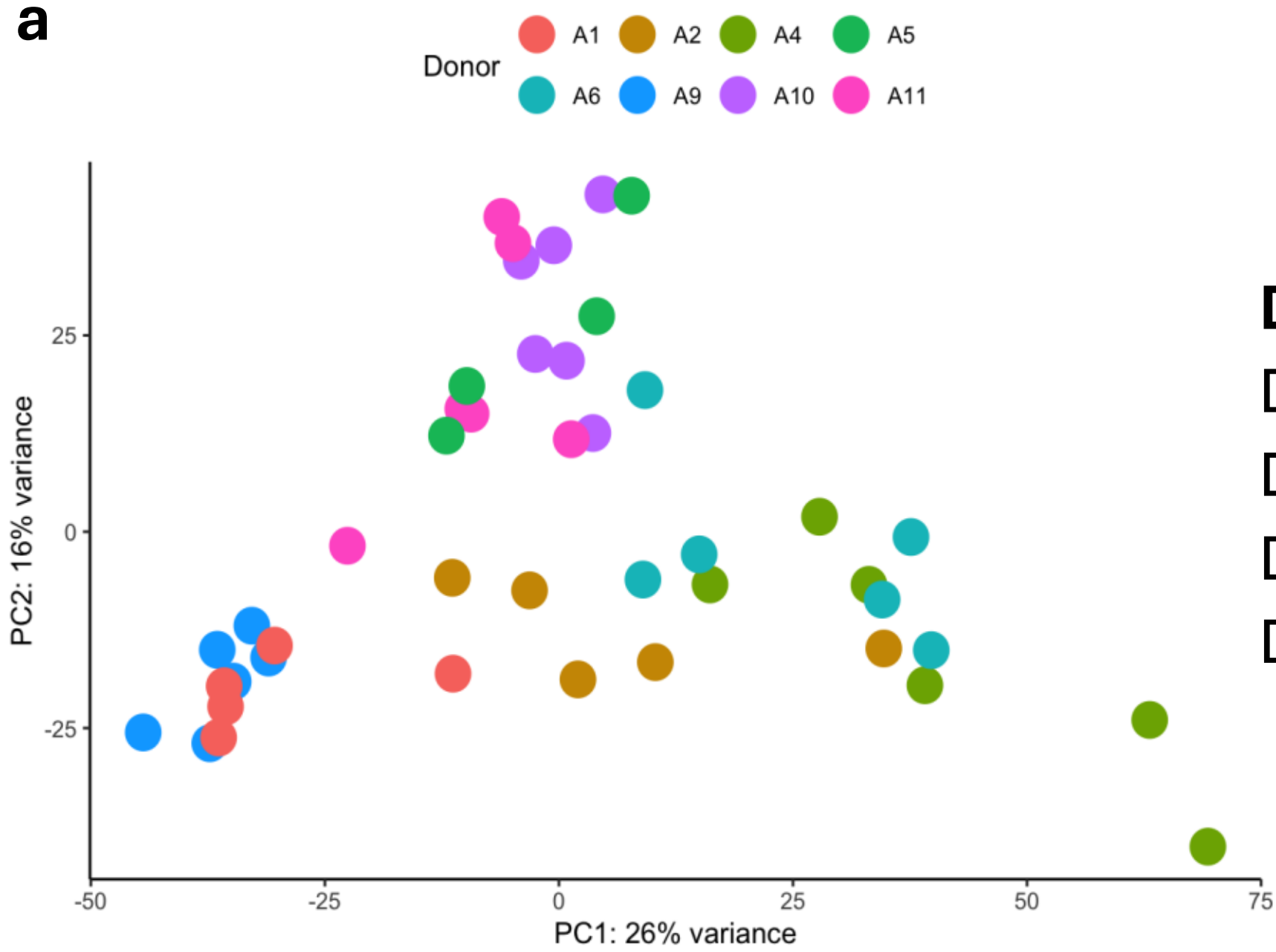

Donor

Donor CH-Index median = 4.23

Donor CH-Index Q1 = 2.59

Donor CH-Index Q3 = 7.93

Donor CH-Index IQR = 5.34

b

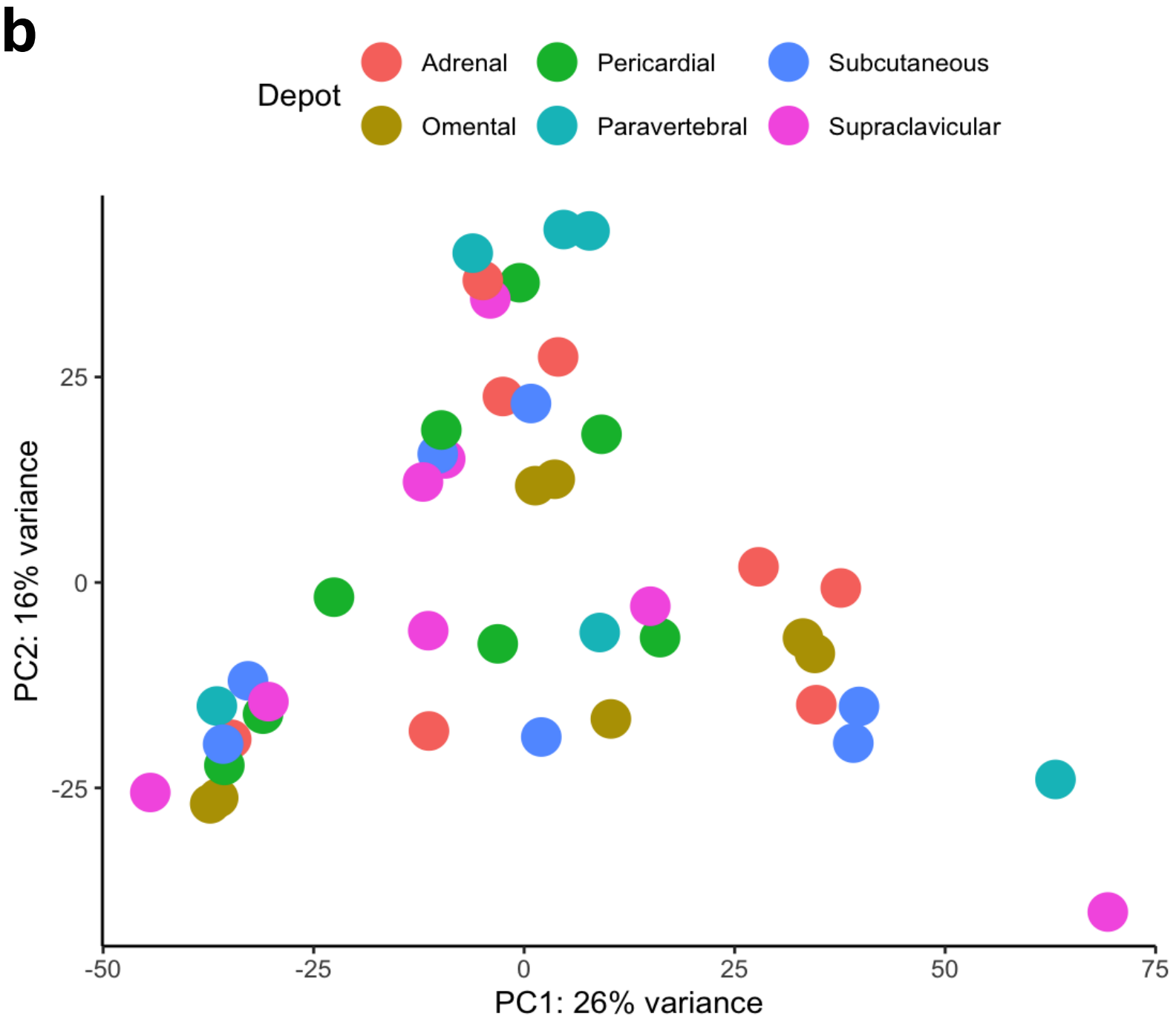

Depot

Depot CH-Index median = 0.49

Depot CH-Index Q1 = 0.35

Depot CH-Index Q3 = 0.71

Depot CH-Index IQR = 0.36

PCA analyses with samples colored by **a** depot and **b** donor. The donor cohort more closely matches the mouse cohort with the removal of female human donors (Donors 3, 7, and 8) and any human donors whose cause of death was not a malignancy (Donors 3 and 7).

Supplementary Figure 8 Impact of long noncoding RNAs and batch correction on clustering.

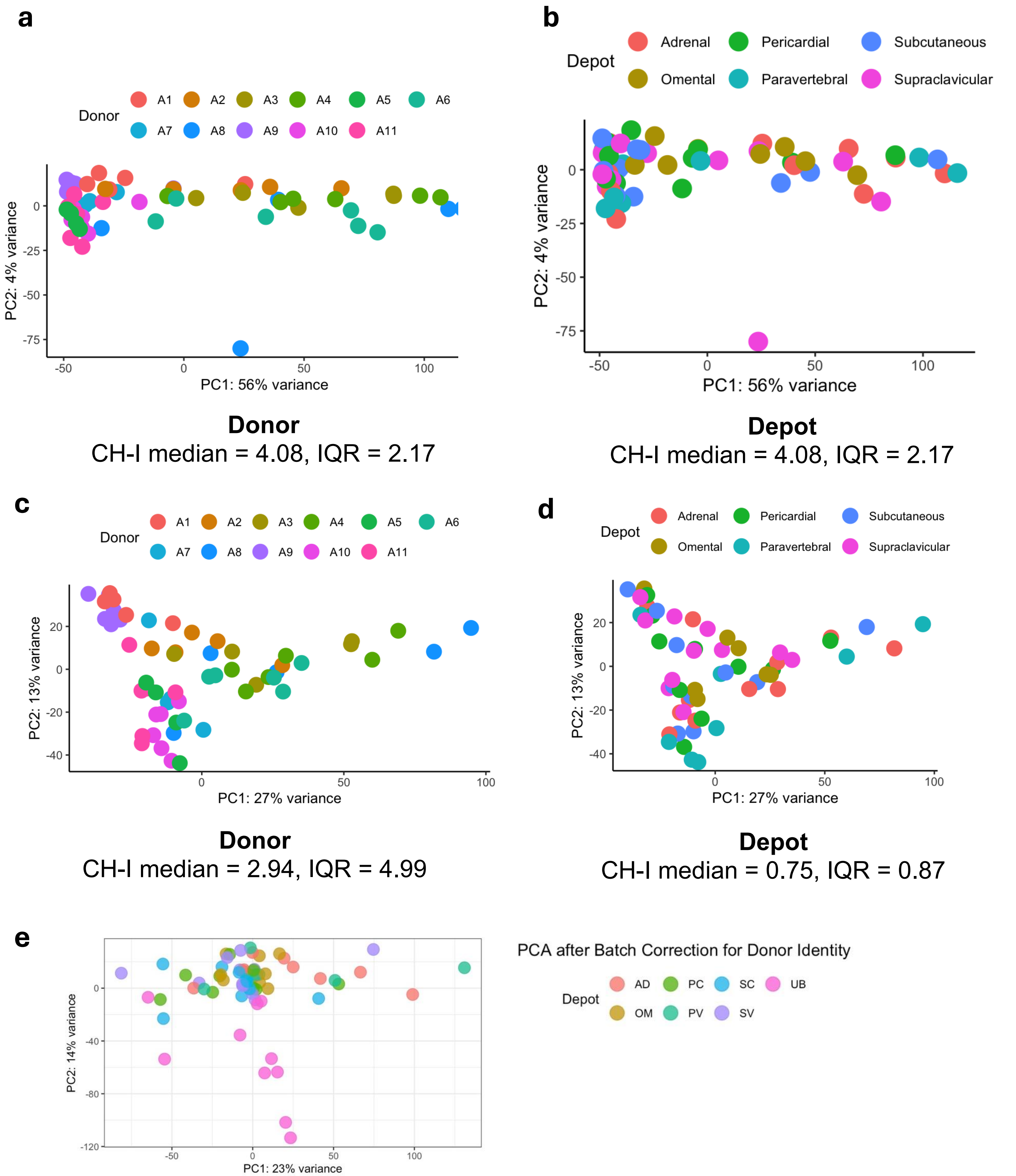

PCA analyses (**a**-**d**) with samples colored by (**a**) and (**c**) donor or (**b**) and (**d**) depot. a and b are PCAs using only lncRNA genes, and c and d are subsetted to exclude all lncRNA genes. Batch correction for (**e**) donor identity.

Supplementary Figure 9 Donors cluster more closely than depots with different gene combinations.

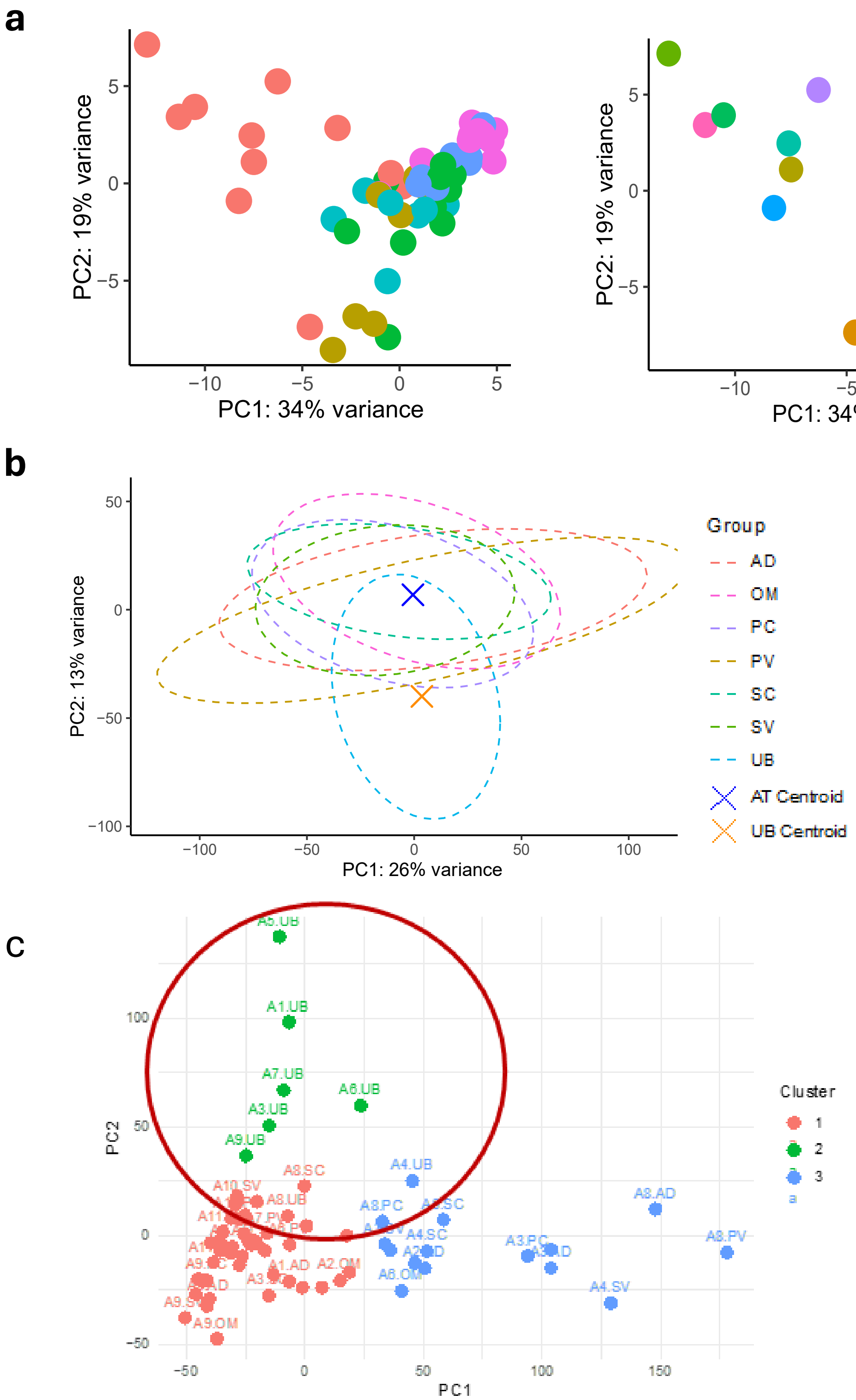

PCA analyses with samples colored by depot (left) and donor (right): (**a**) most variable genes across depots with samples colored by depot (left) and donor (right). (**b**) PCA comparing adipose tissue (AT) to urinary bladder (UB) control using the complete gene list. The crosses represent the centroids; orange for UB; and navy blue for adipose tissue depots. Dotted ellipses indicate 95% confidence interval for all points from a given depot with the red cross representing. (**c**) Unsupervised k-clustering of UB and AT samples with k = 3. Half of the UB samples form a distinct green cluster, while the remaining UB samples localize at the periphery of adipose-dominated clusters. The red circle encompasses all the UB samples.

Supplementary Figure 10 Sample prediction from a training sample using a specific donor or mouse results in the opposite result of using the subcutaneous depot in the training sample.

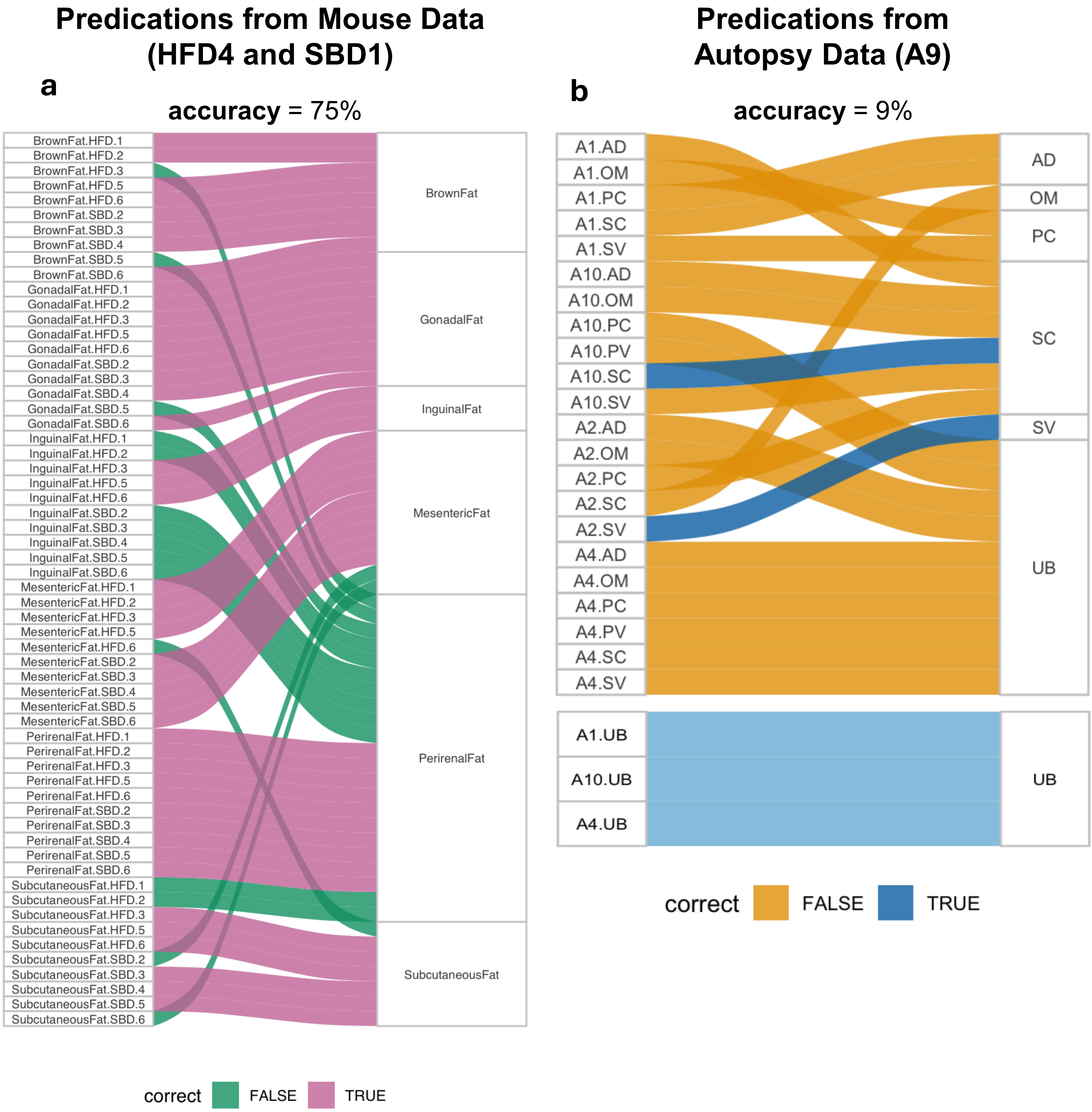

(a) Sankey diagram of a Random Forest analysis using mouse microarray data, with HFD4 and SBD1 mice as the training set, achieved 75% accuracy in predicting adipose depot origin. **(b)** Random Forest model trained on donor A9 data achieved 20% accuracy in predicting depot identity for sample from donors 1, 2, 4, 10. When only considering adipose depot predictions, the model achieved a 9% accuracy rate.

Supplementary Figure 11 Sample prediction on SC samples using withheld visceral (omental) samples.

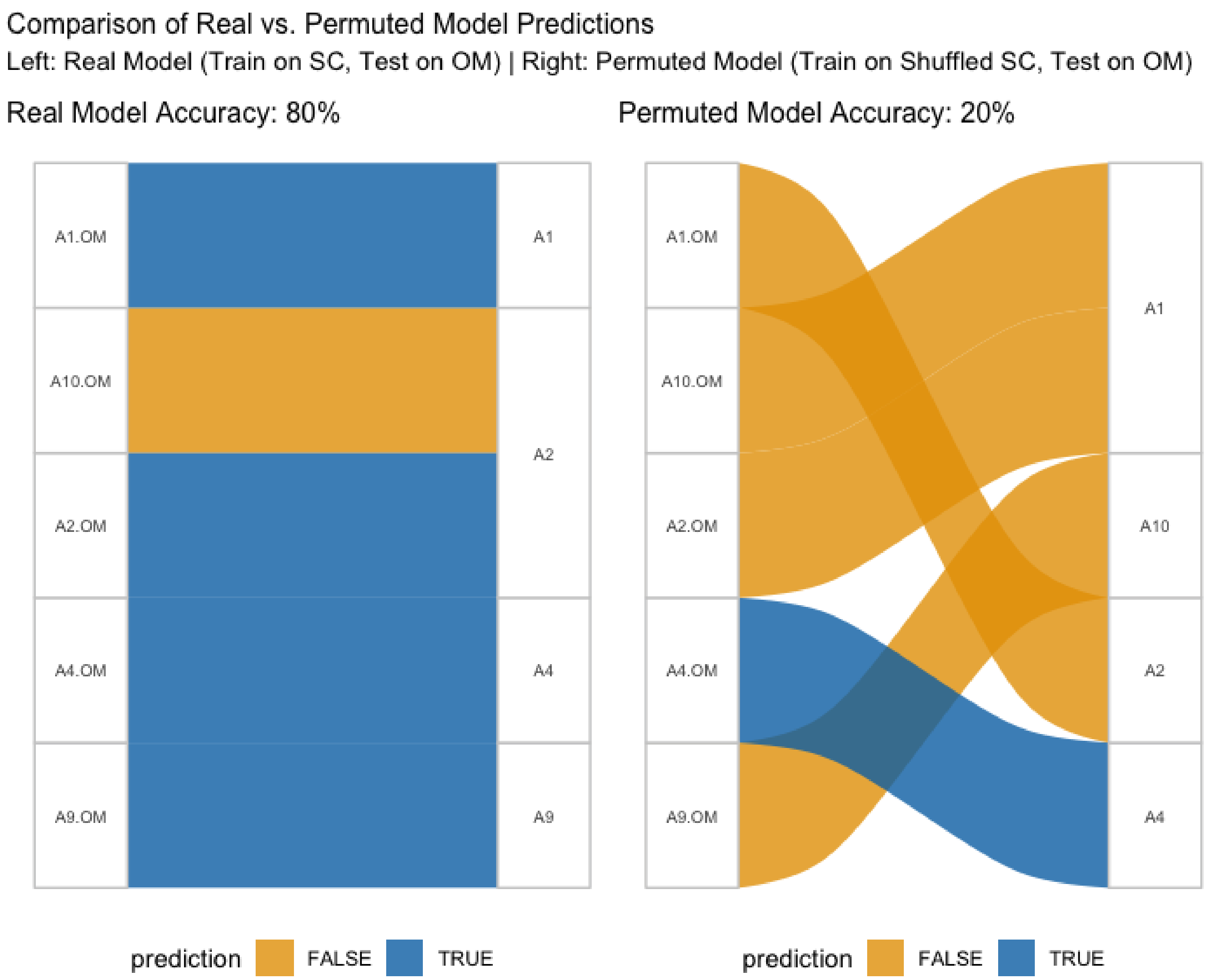

(a) Sankey diagram of a Random Forest analysis using real human donor data and mouse microarray data, with HFD4 and SBD1 mice as the training set, achieved 75% accuracy in predicting adipose depot origin. (**b**) Sankey diagram of a Random Forest analysis using a permuted model.

Supplementary Figure 12 leave-one-donor-out cross-validation (LODO-CV) to assess the model's ability to generalize to new individuals

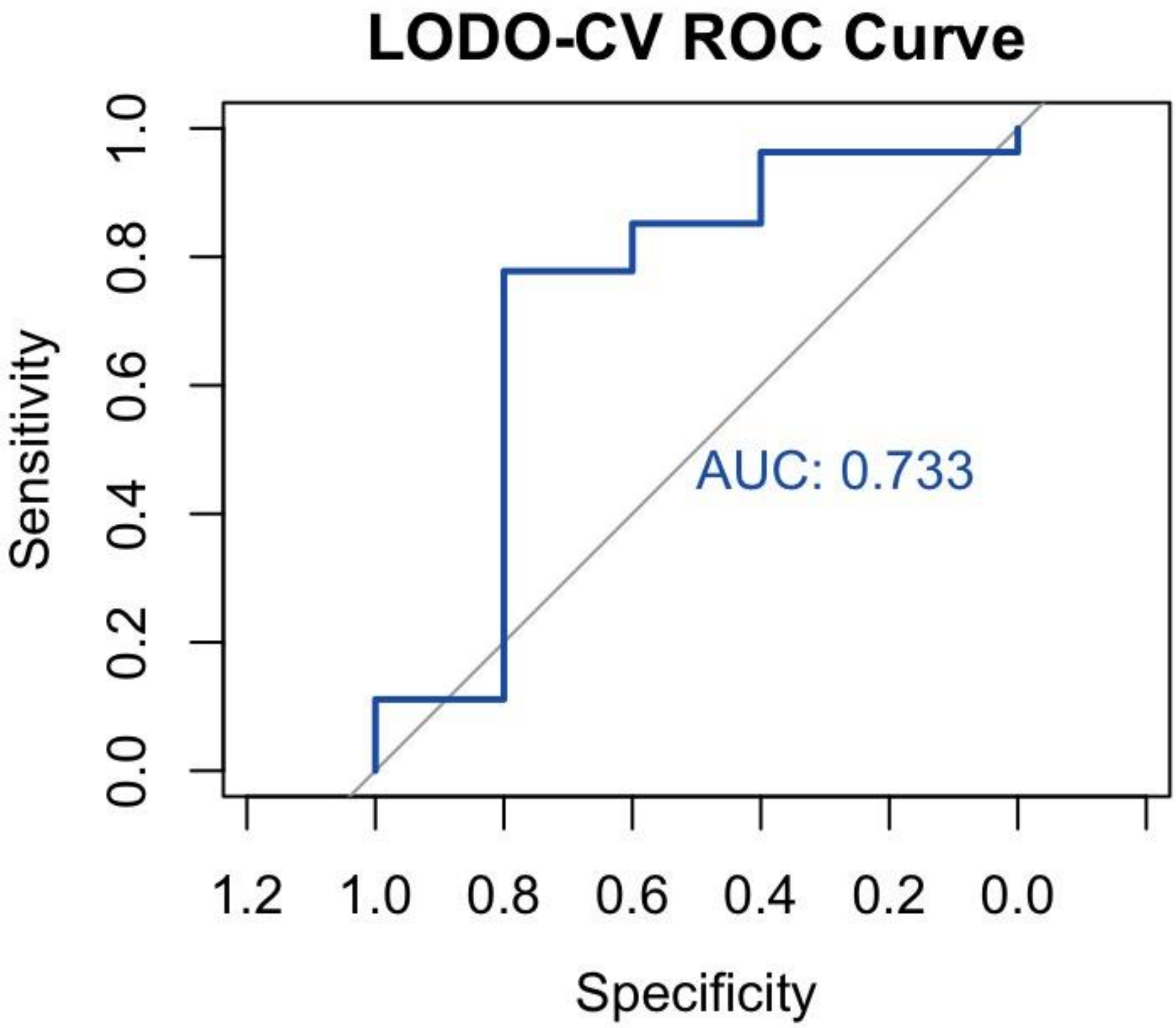

Supplementary Table 1 Donor characteristics and collected tissues.

**Case Code**	**A1**	**A2**	**A3**	**A4**	**A5**	**A6**	**A7**	**A8**	**A9**	**A10**	**A11**
**Sex**	M	M	F	M	M	M	F	F	M	M	M
**BMI (kg*m^{-2})**	25.9	22.4	24.5	25.9	17.9	26.9	21.2	34.3	29.3	22.7	16.8
**Cause of Death**	M	M	I	M	M	M	M	M	M	M	M
**Postmortem Interval (h)**	36.9	12.0	22.5	7.4	72.0	16.0	96.0	55.2	40.0	11.5	48.0
**Tissue Collected**											
Subcutaneous Fat (SC)	+	+	+	+		+	+	+	+	+	+
Omental Fat (OM)	+	+	+	+		+			+	+	+
Pericardial Fat (PC)	+	+	+	+	+	+		+	+	+	+
Adrenal Fat (AD)	+	+	+	+	+	+	+	+	+	+	+
Paravertebral Fat (PV)				+	+	+	+	+	+	+	+
Supraclavicular Fat (SV)	+	+	+	+	+	+	+	+	+	+	+
Urinary Bladder (UB)	+		+	+	+	+	+	+	+	+	+

Cause of Death (CoD)

**M**	Malignancy
**I**	Immune

Demographics of study participants including case number, sex, age, cause of death (CoD), and tissue collection status for each depot. Plus (+) signs indicate successful tissue collection and processing from the specified depot.

Supplementary Table 2 Calinski-Harabasz Index scores for mouse tissue clusters are significantly higher than for individual mouse clusters.

**tissue**	**CH-Index**
BrownFat	50.271
GonadalFat	3.720
InguinalFat	4.277
MesentericFat	65.279
PerirenalFat	3.329
SubcutaneousFat	2.628
median =	**3.999**
Q1 =	**3.427**
Q3 =	**38.773**
IQR =	**35.346**

**mice**	**CH-Index**
SBD.1	0.516
SBD.2	0.042
SBD.3	0.074
SBD.4	0.124
SBD.5	0.168
SBD.6	0.006
HFD.1	0.498
HFD.2	0.824
HFD.3	0.246
HFD.4	0.329
HFD.5	0.320
HFD.6	0.516
median =	**0.283**
Q1 =	**0.112**
Q3 =	**0.502**
IQR =	**0.391**

Calinski-Harabasz Index median, Q1, Q3, and IQR for adipose tissue depot clusters calculated using the top 5,000 genes (**left**). Calinski-Harabasz Index median, Q1, Q3, and IQR for individual mouse clusters calculated using the top 5,000 genes (**right**).

Supplementary Table 3 Calinski-Harabasz Index scores for depot clusters are significantly lower than that for donor clusters.

Depots	CH-Index
SC	0.831
OM	0.298
PC	0.265
AD	1.159
PV	1.631
SV	0.379
median =	**0.605**
Q1 =	**0.318**
Q3 =	**1.077**
IQR =	**0.759**

Donors	CH-Index
A1	5.756
A2	0.318
A3	3.329
A4	7.323
A5	2.039
A6	1.609
A7	0.847
A8	7.028
A9	8.938
A10	4.581
A11	3.012
median =	**3.329**
Q1 =	**1.824**
Q3 =	**6.392**
IQR =	**4.568**

Calinski-Harabasz Index median, Q1, Q3, and IQR for adipose depot clusters calculated calculated using the top 5,000 genes (**left**). Calinski-Harabasz Index median, Q1, Q3, and IQR for adipose donor clusters calculated calculated using the top 5000 genes (**right**).

Supplementary Table 4 The mean Calinski-Harabasz Index score between the depots is less than the mean Calinski-Harabasz Index score between the donors using any combination of genes except when explicitly selecting for depot clustering genes.

PCA	Donors				Depots				p-value (Mann Whit-u)
	Median CH-Index	CH-Index Q1	CH-Index Q3	CH-Index IQR	Median CH-Index	CH-Index Q1	CH-Index Q3	CH-Index IQR	
Top 5,000 Genes	3.329	1.824	6.392	4.568	0.605	0.318	1.077	0.759	0.007
Without Immune Genes	3.505	2.150	4.876	2.726	0.469	0.417	0.899	0.482	6.46E-04
Only Immune Genes	2.713	1.105	6.548	5.443	0.461	0.156	1.502	1.346	0.048
Only Adipogenic Genes	3.177	1.317	6.719	5.402	0.708	0.181	1.050	0.868	0.020
Without Adipogenic Genes	3.335	1.841	6.350	4.510	0.600	0.319	1.078	0.760	0.005
Only Brown (ATCC) Genes	3.141	1.420	6.456	5.036	0.708	0.192	1.138	0.946	0.010
Without Brown (ATCC) Genes	3.218	1.793	6.122	4.329	0.625	0.336	1.010	0.673	0.010
Only miRNA Genes	3.720	3.193	5.102	1.909	0.810	0.293	1.033	0.740	0.002
Random Sample 1	3.341	1.586	5.785	4.199	0.780	0.254	1.253	0.999	0.037
Random Sample 2	3.296	1.377	5.625	4.248	0.550	0.254	1.121	0.867	0.010
Random Sample 3	3.126	1.641	5.887	4.247	0.570	0.309	1.075	0.766	0.027
Top Variability in Depots	0.377	0.278	1.356	1.078	3.496	1.857	7.707	5.850	0.005

Comparative analysis of PCAs generated using different gene subsets, showing mean depot Calinski-Harabasz Index, mean donor Calinski-Harabasz Index, and two-sided Mann-Whitney U test p-values for each unique gene combination.
