# Supplementary material for: Transcriptomic profiling of human brown and white adipose tissue depots
Source: Nat Commun. 2026 Jul 29;17:9181. doi: 10.1038/s41467-026-76065-7 (PMC13522497; doi:10.1038/s41467-026-76065-7)
Supplement: Supplementary file 4 [file 41467_2026_76065_MOESM2_ESM.pdf]

Description of Additional Supplementary Files

File Name: Supplementary Data 1

Description: Tyrosine hydroxylase (TH) immunofluorescence in (**a**) SC, PV, and AD depots with the AD depot as a primary antibody control. Scale bar depicts 50 µm. Each bar represents 1 subject (#9); each point represents 1 image. Error bars are SEM of the n=4 points. * $p < 0.05$, ** $p < 0.01$, Tukey's multiple comparison test, two-sided. qPCR analysis of (**b**) *DIO2* and (**c**) *PPARGC1A* (two thermogenic gene markers). Gene expression data were normalized to ACTIN, the SC depot, and then log-transformed and analyzed using a mixed-effects analysis. For both, n = 10 (SK), 10 (SC), 8 (OM), 10 (PC), 8 (PV), 11 (AD), 11 (SV). Specific *p*-values for the comparisons SK vs SC: 0.0108; SC vs PC: 0.0129; SK vs SV: 0.0020 (**d**) expression matrix depicting expression levels of adipocyte-associated genes. The color scale is based on the log10 fold change, normalized to Actin then to reference SC depot.

File Name: Supplementary Data 2

Description: MA plots visualizing the differential expression between subcutaneous (SC) and omental (OM) (**top left**), pericardial (PC) (**top right**), adrenal (AD) (**bottom left**), and supraclavicular (**bottom right**) depots relative to the subcutaneous depot. Genes with a statistically significant adjusted *p*-value (< 0.05) are highlighted in teal.

File Name: Supplementary Data 3

Description: Deconvolution to determine the presence of structural Wnt-regulated adipose tissue-resident (SWAT), adipogenic, and progenitor cell types.

File Name: Supplementary Data 4

Description: Dot plot analysis showing representative Gene Ontology (GO) pathways enriched in omental (OM) (**top left**), pericardial (PC) (**top right**), adrenal (AD) (**bottom left**), and supraclavicular (SV) (**bottom right**) depots relative to the subcutaneous depot.

File Name: Supplementary Data 5

Description: Violin plot of the top 5000 differentially expressed genes showing percentage variance accounted for (**a**) a mouse model with only tissue and donors as random effect variables; (**b**) in a human autopsy model with only donors and depots as random effect variables; and (**c**) in a human autopsy model with donors, depots, and sex as random effect variables and BMI, RIN, post-mortem interval, and age as fixed

effect variables. Median is shown by central dot in grey bar indicating interquartile range (25th to 75th percentile); whiskers show min/max values.

File Name: Supplementary Data 6

Description: PCA analyses with samples colored by depot (left) and donor (right): (**a)** all adipogenic genes; (**b)** excluding immune function-related genes; (**c)** grouped by donor age bins, where bins represent standard deviations from mean donor age.

File Name: Supplementary Data 7

Description: PCA analyses with samples colored by **a** depot and **b** donor. The donor cohort more closely matches the mouse cohort with the removal of female human donors (Donors 3, 7, and 8) and any human donors whose cause of death was not a malignancy (Donors 3 and 7).

File Name: Supplementary Data 8

Description: PCA analyses (**a-d**) with samples colored by (**a**) and (**c**) donor or (**b**) and (**d**) depot. a and b are PCAs using only lncRNA genes, and c and d are subsetted to exclude all lncRNA genes. Batch correction for (**e**) donor identity.

File Name: Supplementary Data 9

Description: PCA analyses with samples colored by depot (left) and donor (right): (**a)** most variable genes across depots with samples colored by depot (left) and donor (right). (**b)** PCA comparing adipose tissue (AT) to urinary bladder (UB) control using the complete gene list. The crosses represent the centroids; orange for UB; and navy blue for adipose tissue depots. Dotted ellipses indicate 95% confidence interval for all points from a given depot with the red cross representing. (**c)** Unsupervised k-clustering of UB and AT samples with k = 3. Half of the UB samples form a distinct green cluster, while the remaining UB samples localize at the periphery of adipose-dominated clusters. The red circle encompasses all the UB samples.

File Name: Supplementary Data 10

Description: (a) Sankey diagram of a Random Forest analysis using mouse microarray data, with HFD4 and SBD1 mice as the training set, achieved 75% accuracy in predicting adipose depot origin. (**b)** Random Forest model trained on donor A9 data achieved 20% accuracy in predicting depot identity for sample from donors 1, 2, 4, 10. When only considering adipose depot predictions, the model achieved a 9% accuracy rate.

File Name: Supplementary Data 11

Description: **(a)** Sankey diagram of a Random Forest analysis using real human donor data and mouse microarray data, with HFD4 and SBD1 mice as the training set, achieved 75% accuracy in predicting adipose depot origin. (**b)** Sankey diagram of a Random Forest analysis using a permuted model.

File Name: Supplementary Data 12

Description: Leave-one-donor-out cross-validation (LODO-CV) to assess the model's ability to generalize to new individuals.
